# Signatures of proteomics and glycoproteomics revealed liraglutide ameliorates MASLD by regulating specific metabolic homeostasis in mice

**DOI:** 10.1016/j.jpha.2025.101273

**Published:** 2025-03-19

**Authors:** Yuxuan Chen, Chendong Liu, Qian Yang, Jingtao Yang, He Zhang, Yong Zhang, Yanruyu Feng, Jiaqi Liu, Lian Li, Dapeng Li

**Affiliations:** a Key Laboratory of Drug Targeting and Drug Delivery System of the Education Ministry and Sichuan Province, Sichuan Engineering Laboratory for Plant-Sourced Drug and Sichuan Research Center for Drug Precision Industrial Technology, West China School of Pharmacy, Sichuan University, Chengdu, 610041, China; b Department of Laboratory Medicine, West China Hospital, Sichuan University, Chengdu, 610041, China; c Department of Nephrology, Institutes for Systems Genetics, West China Hospital, Sichuan University, Chengdu, 610041, China; d Ninth People's Hospital of Zhengzhou, Zhengzhou, 45000, China

**Keywords:** Liraglutide, Metabolic dysfunction-associated steatotic liver disease, Proteomics, Glycoproteomics, N-glycosylation site, Hydrogel-based delivery

## Abstract

Liraglutide (Lira), a glucagon-like peptide-1 (GLP-1) receptor agonist approved for diabetes and obesity, has shown significant potential in treating metabolic dysfunction-associated steatotic liver disease (MASLD). However, its systematic molecular regulation and mechanisms remain underexplored. In this study, a mouse model of MASLD was developed using a high-fat diet (HFD), followed by Lira administration. Proteomics and glycoproteomics were analyzed using label-free liquid chromatography-tandem mass spectrometry (LC-MS/MS), while potential molecular target analysis was conducted via quantitative real-time polymerase chain reaction (qPCR) and Western blotting. Our results revealed that Lira treatment significantly reduced liver weight and serum markers, including alanine aminotransferase (ALT) and others, with glycosylation changes playing a more significant role than overall protein expression. The glycoproteome identified 255 independent glycosylation sites, emphasizing the impact of Lira on amino acid, carbohydrate metabolism, and ferroptosis. Simultaneously, proteomic analysis highlighted its effects on lipid metabolism and fibrosis pathways. 21 signature molecules, including 7 proteins and 14 N-glycosylation sites (N-glycosites), were identified as potential targets. A Lira hydrogel formulation (Lira@fibrin (Fib) Gel) was developed to extend drug dosing intervals, offering enhanced therapeutic efficacy in managing chronic metabolic diseases. Our study demonstrated the importance of glycosylation regulation in the therapeutic effects of Lira on MASLD, identifying potential molecular targets and advancing its clinical application for MASLD treatment.

## Highlights

• Comprehensive proteomics and glycoproteomics revealed MASLD mechanisms in HFD mice.
• Liraglutide increased glycosylation, identifying 255 independent and impactful sites.
• Liraglutide regulated amino acid and lipid metabolism, ferroptosis, and fibrosis.
• Identified 21 signature molecules offered potential therapeutic targets.
• Lira@Fib Gel improved delivery and efficacy for chronic metabolic disease management.

## Graphical abstract

**Figure:**
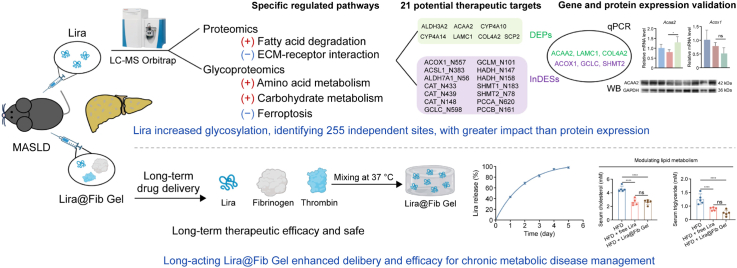

## 1 Introduction

Metabolic dysfunction-associated steatotic liver disease (MASLD) was previously known as non-alcoholic fatty liver disease [1]. It is a major global health issue associated with obesity, type 2 diabetes mellitus (T2DM), metabolic syndrome, and an increased risk of cardiovascular disease [2]. Although lifestyle interventions remain the primary treatment approach, achieving significant weight loss is challenging for most patients [3,4]. Metabolic dysfunction-associated steatohepatitis (MASH) is a progressive form of MASLD. The recent U.S. Food and Drug Administration (FDA) approval of resmetirom for MASH with liver fibrosis has progressed, but further therapeutic options remain urgent [[5], [6], [7]].

Liraglutide (Lira), a glucagon-like peptide-1 (GLP-1) receptor agonist, has been approved for treating diabetes and obesity by FDA [8], and it exhibits promise in improving liver health by reducing hepatic steatosis and inflammation. However, the underlying mechanisms remain controversial and not fully explored. Yang et al. [9] demonstrated the efficacy of Lira via the insulin receptor substrate 2/phosphatidylinositol 3 kinase/protein kinase B (IRS2/PI3K/Akt) signaling pathway. Luo et al. [10] highlighted its role in modulating lipid metabolism and inflammation [10]. Additionally, Lira ameliorates obesity-associated MASLD through sestrin2 and the nuclear factor erythropoietin-2-related factor 2/heme oxygenase 1 (Nrf2/HO-1) pathways, as well as inhibits ferroptosis in T2DM-induced MASLD via adenosine 5'-monophosphate (AMP)-activated protein kinase/acetyl-CoA carboxylase (AMPK/ACC) signaling [11,12]. However, the potential side effects of Lira, including elevated liver enzymes, cholestasis, hepatitis, and even autoimmune hepatitis, raise concerns regarding its safety in treating MASLD [13]. Therefore, a systematic and comprehensive analysis of the molecular regulatory networks, targets, and effects of Lira is urgently required.

Protein glycosylation, particularly N-glycosylation, is integral to cellular processes such as cell adhesion, growth, and signaling. It is notably altered in MASH, emphasizing its essential role in disease progression [14]. Glycoproteomics studies have revealed distinct glycosylation patterns in the MASH serum [15]. Accordingly, we hypothesized that the mechanism of Lira in improving MASLD could be connected to alterations in the *N*-glycoproteome, an underexplored area, highlighting the critical need for further detailed investigation.

Here, we explored the systemic regulatory mechanisms by which Lira improves MASLD, focusing on its impact on N-glycosylation. We provided the first system-level overview of N-glycoproteins and N-glycosylation sites (N-glycosites) in MASLD mouse livers treated with Lira using advanced quantitative proteomic and glycoproteomic analyses. Our findings revealed significant changes in the glycoproteome, with pathway analysis identifying signature molecules behind its therapeutic effects as potential therapeutic targets, advancing our understanding of the role of Lira in MASLD treatment. Additionally, the short half-life of Lira of approximately 12 h necessitates daily injections, which can result in adverse effects such as tissue damage and pain, negatively affecting patient compliance, particularly given the long recovery course of MASLD. To address this, we developed an *in-situ* hydrogel formulation to extend the dosing interval of Lira, offering a simple yet promising solution for clinical applications. This study aimed to enhance the therapeutic potential of Lira and improve patient compliance, thereby advancing its role in MASLD and related metabolic disease management.

## 2 Materials and methods

### 2.1 Materials and reagents

The bicinchoninic acid (BCA) protein assay kit was bought from Beyotime Biotechnology (Shanghai, China). NH_4_HCO_3_ (purity >99%) and urea (UA) (purity >99.5%) were procured from Shanghai Aladdin Bio-Chem Technology Co., Ltd. (Shanghai, China). Proteomic-grade trypsin was supplied by Promega Corporation (Madison, WI, USA). Dithiothreitol (DTT) and iodoacetamide (IAA) were acquired from Sigma Aldrich (St. Louis, MO, USA) for reduction and alkylation. Lira (CAS: 204656-20-2) was provided by HEC Pharma Co., Ltd. (Dongguan, China). High performance liquid chromatography (HPLC)-grade acetonitrile (ACN) was procured from GHTECH (Shantou, China), while formic acid (FA) and trifluoroacetic acid were obtained from Chengdu Chron Chemicals Co., Ltd. (Chengdu, China). Deionized water was purified using a Direct-Q5 ultraviolet (UV) (Merck Ltd., Darmstadt, Germany). Nanosep centrifugal filters (10 kDa) were produced by Pall Corporation (New York, NY, USA). Bovine fibrinogen (Cat. No.: 20430ES03) and bovine thrombin (Cat. No.: 20402ES03) were provided by Yeasen Biotechnology (Shanghai) Co., Ltd. (Shanghai, China). Optimal cutting temperature (OCT) compound (Cat. No.: 4583), total cholesterol (TC) content assay kit (Cat. No.: BC1985), and modified oil red O stain kit (Cat. No.: G1263) were provided by Beijing Solarbio Science & Technology Co., Ltd. (Beijing, China). SteadyPure RNA Extraction Kit (Code No.: AG21024), Evo M-MLV RT Mix Kit with DNA-guided (gDNA) Clean for quantitative polymerase chain reaction (qPCR) Ver.2 (Code No.: AG11728) and SYBR Green Premix Pro Taq HS qPCR Kit (Code No.: AG11718) were bought from Accurate Biology (Changsha, China).

### 2.2 Lira administration and biochemical analysis in MASLD-induced C57/BL6 mice

Specific pathogen-free male C57/BL6 mice (six weeks old, weighing 18 ± 2 g) were acquired from the Beijing Vital River Laboratory Animal Technologies Co., Ltd. (Beijing, China) and housed in a specific pathogen-free environment at 21 ± 1 °C with 60% ± 5% humidity and a 12-h light-dark cycle. All animal experiments were approved by the Institutional Animal Care and Ethics Committee of Sichuan University, China (Approval No.: SCU42-2409-01).

To induce MASLD, the mice were fed a high-fat diet (HFD) (60% kcal fat + 20% kcal sucrose) for three months. Following the MASLD induction, the mice were subcutaneously administered Lira at a dose of 1 mg/kg once daily for seven days. At the end of treatment, whole blood, liver, and white fat tissues were collected.

Blood samples were clotted at room temperature for 4 h, centrifuged at 3,000 rpm for 5 min, and the supernatant was collected, diluted with phosphate-buffered saline (PBS) (1:2, *v*/*v*), aliquoted, and stored at −80 °C. MASLD-related serum biochemical parameters, including alkaline phosphatase (ALP), alanine aminotransferase (ALT), aspartate aminotransferase (AST), low-density lipoprotein (LDL), high-density lipoprotein (HDL), and triglyceride (TG), were analyzed using an automatic biochemical analyzer (COBAS C311, Roche Diagnostics, Mannheim, Germany). COBAS C system reagents (Table S1, Roche Diagnostics) were loaded, and quality control was performed before sample measurement. Aliquoted samples were thawed and analyzed after passing quality control.

The liver and white fat tissues were weighed, and total lipids from the liver were extracted using a dichloromethane-methanol mixture (2:1, *v*/*v*) and then weighed after solvent evaporation. The cholesterol content in the liver was measured using a TC assay kit. Liver tissues were fixed, embedded in OCT compound, and sectioned for histological analysis using a modified oil red O stain kit.

### 2.3 Protein extraction and trypsin digestion for proteomics

For protein extraction, 10 mg of mouse liver tissue was homogenized in 500 μL of lysis buffer (8 M UA in 50 mM ammonium bicarbonate, with a 1% protease inhibitor cocktail). The sample was milled at 1,500 rpm for 20 s, repeated thrice, and lysed on ice for 2 h with intermittent vortexing. The lysate was then homogenized by sonication on ice (6 cycles of 5 s with 20 s intervals). After centrifugation at 16,000 *g* for 20 min at 4 °C, the supernatant was collected, and the protein concentration was measured using a BCA kit [16]. For trypsin digestion, 300 μg protein was subjected to filter-assisted sample preparation. The lysate was treated with DTT and IAA for reduction and alkylation, followed by overnight trypsin digestion at 37 °C [17]. The resulting peptides were split for proteomic and N-glycoproteomic analyses, with proteomics peptides desalted using Pierce C18 Spin Tips.

### 2.4 N-glycopeptides enrichment and deglycosylation

*N*-glycopeptides were enriched following a previously reported protocol [18]. Briefly, lyophilized peptides were dissolved in loading buffer (ACN:H_2_O:trifluoroacetic acid = 95:4.9:0.1, *v*/*v*/*v*) and incubated with hydrophilic interaction chromatography (HILIC) material at 37 °C for 60 min. After centrifugation at 14,000 *g* for 5 min, the supernatant was discarded, and the sample was washed with washing buffer (ACN:H_2_O:FA = 85:15:0.5, *v*/*v*/*v*). Elution was performed using an eluting buffer (ACN/H_2_O/FA = 30:70:0.5, *v*/*v*/*v*), repeated thrice, and the combined eluates were lyophilized. The glycopeptides were then solubilized in 10 mM NH_4_HCO_3_ and deglycosylated with PNGase F at 37 °C for 16 h, followed by freeze-drying and storage at −80 °C.

### 2.5 Nano liquid chromatography-tandem mass spectrometry (LC-MS/MS) analysis

The lyophilized peptides were resuspended in 0.1% FA and analyzed alongside the glycopeptides using nano LC-MS/MS. Peptide separation was performed using an EASY-nLC 1200 system (Thermo Fisher Scientific Inc., Waltham, MA, USA) equipped with a capillary column (75 μm × 300 mm) packed with C_18_ resin (1.9 μm; Dr. Maisch, Tübingen, Germany). The mobile phases comprised 0.1% FA in water (A) and 0.1% FA in 80% ACN (B). The elution gradient was set over 78 min, starting from 3% to 100% B at a 300 nL/min flow rate. The peptides were then analyzed on an Orbitrap Exploris™ 480 mass spectrometer (Thermo Fisher Scientific Inc.) in positive ion mode using the Xcalibur 4.2 data acquisition system. The primary MS scan ranged from *m*/*z* 350 to 1,500 with a resolution of 60,000, and the normalized automatic gain control (AGC) target was set to 300% with a maximum injection time of 50 ms. Data were acquired in a data-dependent acquisition mode. The top 10 abundant precursor ions from the primary scan were selected for further analysis using high-energy collisional dissociation with a resolution of 15,000. The isolation window was set to *m*/*z* 1.6, the normalized collision energy to 30%, and the AGC target to 75%, with a maximum injection time of 22 ms.

### 2.6 Data statistics and bioinformatic analysis

Raw data were processed using Fragpipe software (version 20.0) for database searching against the mouse SwissProt (version 20230709; 21,866 entries) and reverse decoy databases. The parameters included a precursor mass tolerance of ±20 ppm, fragment mass tolerance of ±20 ppm, up to two missed tryptic cleavages, and fixed carbamidomethylation on cysteines. Variable modifications include methionine oxidation and N-terminal acetylation. A minimum peptide length of seven amino acids and a false discovery rate of <1% were applied at the protein and peptide levels [19]. *N*-glycopeptides were specifically analyzed using MaxQuant (version 2.4.2.0) with trypsin/P as the cleavage enzyme, allowing for up to two missed cleavages. The mass tolerance for the first search was set at 20 and 4.5 ppm for the main search, with a fragment ion mass tolerance of 0.02 Da. Fixed modifications included carbamidomethylation, while variable modifications included N-terminal acetylation, methionine oxidation, and deamidation 18O (N). The N−X–S/T/C (X ≠ proline) motif, characteristic of N-glycosites, was filtered specifically during the analysis. Glycopeptide abundance was quantified using normalized spectral protein intensity (label-free quantification (LFQ) intensity) [14]. Subsequent data filtering, *t*-tests, and multi-sample analysis of variance (ANOVA) were performed using Perseus (version 2.0.10.0). Data interpolation was performed using NAguideR [20] and normalization using NormalyzerDE [21]. Functional annotation, including Gene Ontology (GO) and Kyoto Encyclopedia of Genes and Genomes (KEGG) analysis, was completed using R (version 4.3.2). Protein-protein interaction (PPI) analysis was conducted with the String database (confidence 0.700) and visualized using Cytoscape (version 3.10.1). GraphPad Prism 9 was used for data analysis and visualization.

### 2.7 Quantitative real-time PCR and Western blotting assay

Mouse livers (20 mg each approximately) were weighed for RNA and protein extraction. Total RNA was isolated using a SteadyPure RNA Extraction Kit (Code No.: AG21024; Accurate Biology) and assessed for concentration and quality. RNA was then reverse transcribed into complementary DNA (cDNA) using the Evo M-MLV RT Mix Kit with gDNA Clean for qPCR Ver.2 (Accurate Biology). Gene expression was quantified using the SYBR Green Premix Pro Taq HS qPCR Kit (Accurate Biology) with the 2^−ΔΔ*Ct*^ method and normalized to β-actin. The primer sequences listed in Table S2. Real-time PCR was performed using a QuantStudio 3 Real-Time PCR System (Thermo Fisher Scientific Inc.). The amplification program was as follows: 95 °C for 30 s, and 40 cycles at 95 °C for 5 s and 60 °C for 30 s. For protein extraction, liver tissues were lysed in pre-cooled radioimmune precipitation assay (RIPA) buffer, and the proteins were quantified using a BCA kit. Equal amounts of protein were denatured at 90 °C for 15 min. Proteins were separated via sodium dodecyl sulfate-polyacrylamide gel electrophoresis (SDS-PAGE) (90 V for 30 min and 120 V for 60 min) and transferred to a 0.45-μm polyvinylidene fluoride (PVDF) membrane. Blocking was performed for 15 min, followed by overnight incubation with the primary antibody at 4 °C. After tris buffered saline with Tween-20 (TBST) washes, samples were incubated with horseradish peroxidase (HRP)-secondary antibody for 1 h, and bands were detected with EZ electrochemiluminescence (ECL) Pico substrate and quantified using the ImageJ software. The primary antibodies used in our study were as follows: laminin subunit gamma-1 (LAMC1) (1:8000, 67706-1-Ig; Proteintech Group, Inc., Wuhan, China), collagen type IV alpha-2 chain (COL4A2) (1:5000, 55131-1-AP; Proteintech Group, Inc.), acetyl-CoA acyltransferase 2 (ACAA2) (1:5000, 11111-1-AP; Proteintech Group, Inc.), glutamate-cysteine ligase catalytic subunit (GCLC) (1:6000, 12601-1-AP; Proteintech Group, Inc.), glyceraldehyde 3-phosphate dehydrogenase (GAPDH) (1:100000, 60004-1-Ig; Proteintech Group, Inc.), and α-tubulin (1:6000, PTM-5001; PTM BIO, Hangzhou, China). Anti-mouse (1:10,000, RS0001; Immunoway, Plano, TX, USA) or anti-rabbit (1:10,000, RS0002; Immunoway) HRP-conjugated IgG was used as secondary antibodies.

### 2.8 Formulation of Lira-loaded hydrogel with therapeutic efficacy in long-term MASLD treatment

The Lira-loaded hydrogel (Lira@Fib Gel) was prepared by adding 10 μL of thrombin (500 U/L) and Lira (0.5 mg) to 1 mL of Fib solution (50 mg/mL), followed by incubation at 37 °C to allow for gelation. The rheological properties and micromorphology of the Lira-loaded hydrogel were characterized. For drug release studies, 1 mL of the hydrogel was immersed in 2 mL of PBS (pH 7.4) at 37 °C, with samples collected at predefined intervals to analyze Lira concentrations using HPLC (1260 Infinity II; Agilent Technologies, Santa Clara, CA, USA).

*In vivo*, MASLD model mice (*n* = 5) were administered injections of hydrogel (equivalent to 4 mg/kg Lira every four days) over 28 days. The control group received daily injections of free Lira (1 mg/kg/day). At the study endpoint, MASLD-related blood biochemical parameters, liver and white fat weights, and organ injury markers (total protein (TP), albumin (ALB), lactate dehydrogenase (LDH), creatine kinase myocardial band (CKMB), amylase, creatinine, and UA), were assessed.

## 3 Results

### 3.1 Short-term Lira treatment ameliorated MASLD in mice

**Fig. 1:**
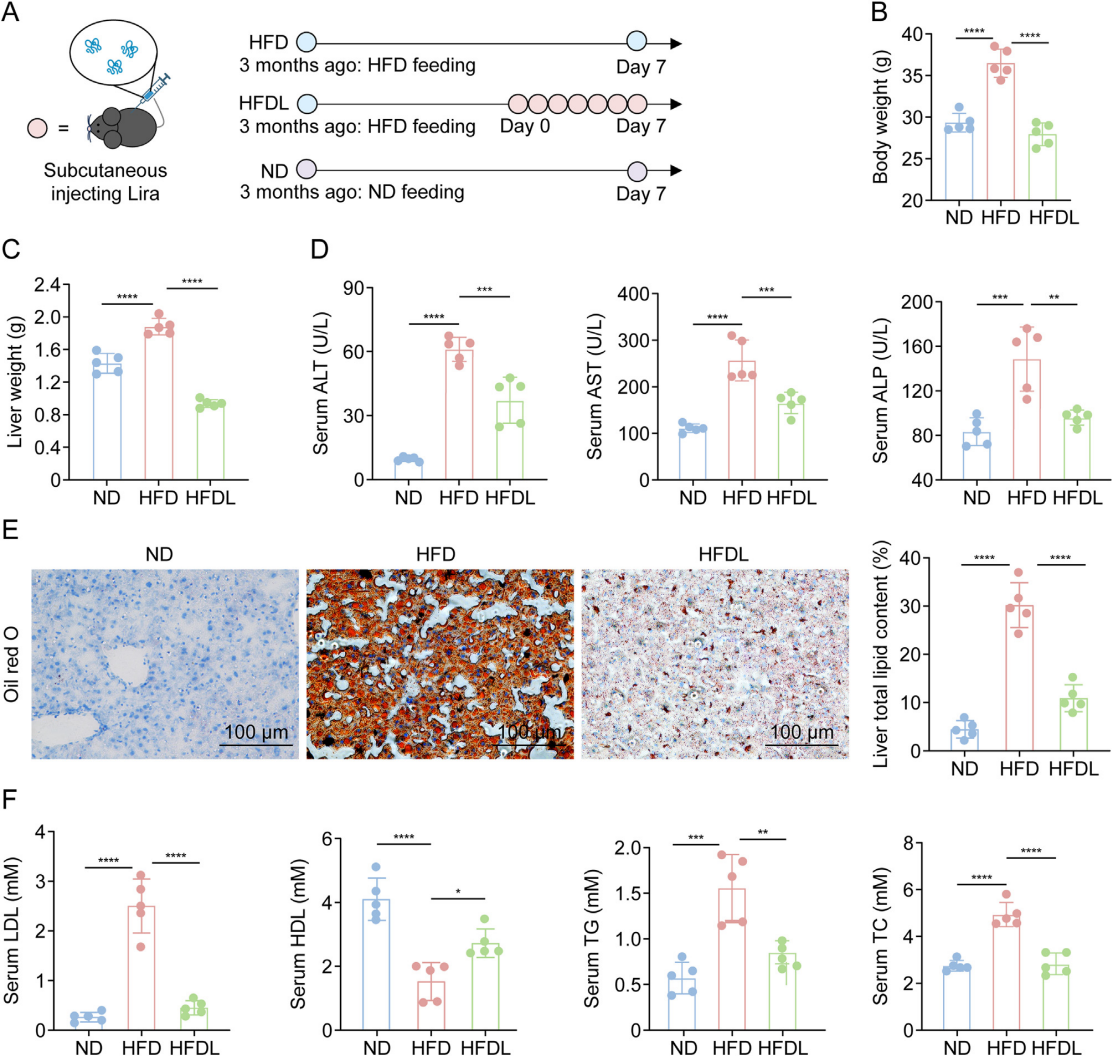
Therapeutic effects of short-term daily liraglutide (Lira) administration in a male C57/BL6 mouse metabolic dysfunction-associated steatotic liver disease (MASLD) model. (A) Treatment schedule. (B, C) Body weight (B) and liver weight (C) at the endpoint. (D) Indicators of liver injury, including alanine aminotransferase (ALT), aspartate aminotransferase (AST), and alkaline phosphatase (ALP) levels. (E) Lipid accumulation in the liver, assessed by oil red O staining and quantification of lipid content in the liver. (F) Serum levels of low-density lipoprotein (LDL), high-density lipoprotein (HDL), triglyceride (TG), and total cholesterol (TC). Data is presented as mean ± standard deviation (SD) (*n* = 5 animals per group). Statistical significance was calculated using one-way analysis of variance (ANOVA) with Tukey's multiple comparisons. Blue circles: HFD-only mice; pink circles: HFD + liraglutide treatment; purple circles: normal diet mice. ^∗^*P* < 0.05, ^∗∗^*P* < 0.01, ^∗∗∗^*P* < 0.001, and ^∗∗∗∗^*P* < 0.0001. HFD: high-fat diet; HFDL: HFD + Lira; ND: normal diet.

To induce MASLD symptoms, male C57/BL6 mice were fed an HFD for three months (Fig. 1A), resulting in a significant weight gain (Fig. 1B). Compared to the dark red livers of healthy controls (ND group), HFD-fed mice displayed pale, swollen livers (Figs. 1C and S1A). Serum ALT, AST, and ALP levels were significantly elevated in HFD-fed mice, indicating hepatocellular damage (Fig. 1D). Oil Red O staining revealed extensive hepatic lipid droplets, highlighting severe fat deposition and steatosis (Figs. 1E and S1B). HFD also induced dysregulation of systemic lipid metabolism, as evidenced by increased epididymal white fat accumulation (Fig. S1C), elevated serum LDL, TG, and TC levels, and reduced HDL levels (Fig. 1F). These results underscore the extensive metabolic and hepatic effects of HFD.

Subcutaneous Lira injection significantly improved MASLD symptoms in mice after one week (Fig. 1A). Body weight returned to normal (Fig. 1B), and the livers appeared deep red with reduced size, intermediate between the healthy and HFD states (Fig. S1A). Liver weight and liver-to-body weight ratio confirmed a marked reduction in HFD-induced swelling (Fig. 1C). Serum ALT, AST, and ALP levels decreased significantly, indicating hepatoprotective effects of Lira (Fig. 1D). The number of lipid droplets in the liver was reduced. However, some intracellular lipid deposition remained (Figs. 1E and S1B), consistent with liver morphology (Fig. S1A). Lira promoted fat degradation and lipid metabolism, reducing epididymal white fat to healthy levels (Fig. S1C) and improving serum lipid profiles, with lower LDL, TG, and TC levels and restored HDL levels (Fig. 1F).

### 3.2 Global proteomic and glycoproteomic profiles of MASLD liver following Lira intervention

**Fig. 2:**
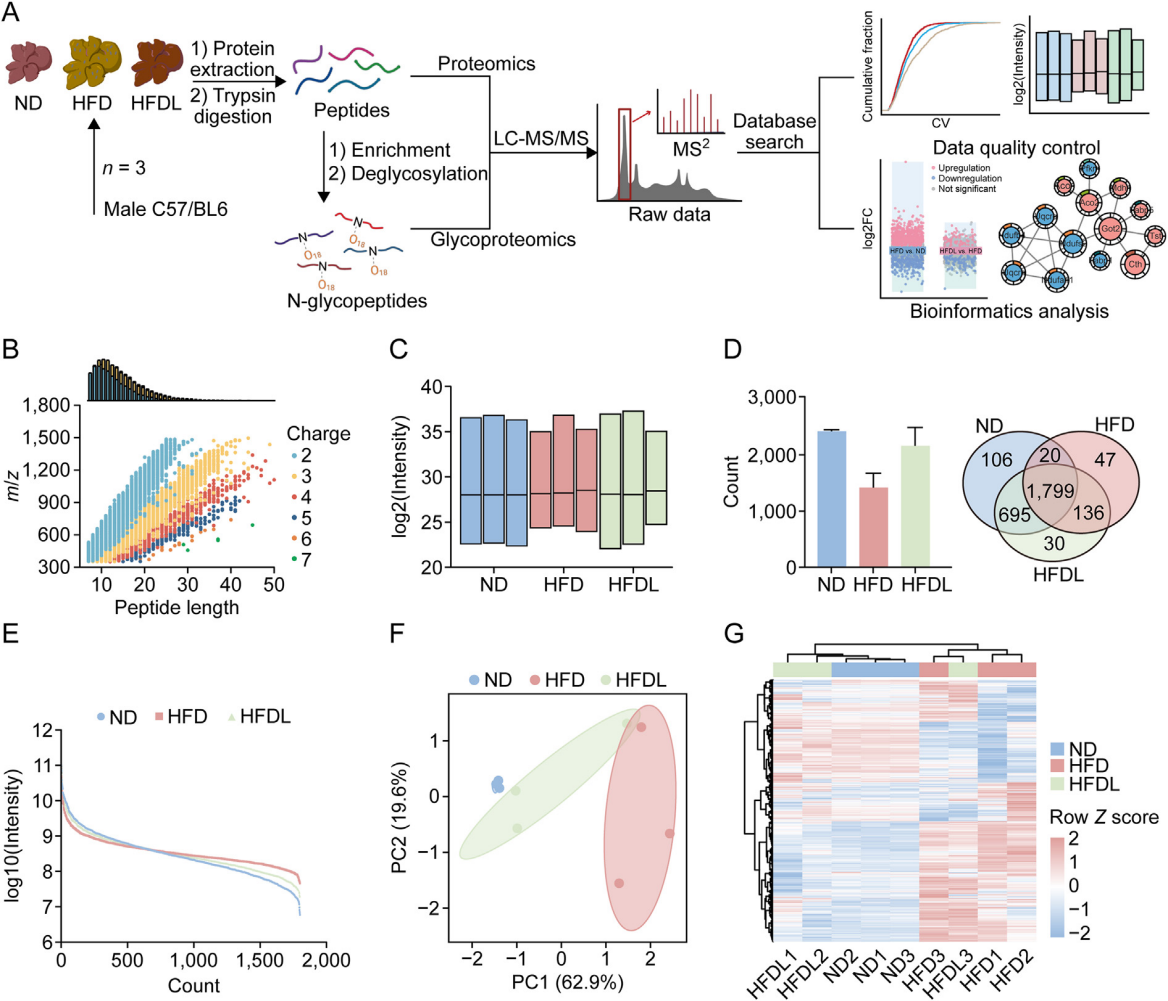
Workflow and overview of mouse liver proteomics results. (A) Workflow for proteomic and glycoproteomic analysis. (B, C) Quality control analysis of the global liver proteome, including distribution of peptide lengths across samples (B) and log2(intensity) distribution (C) in the indicated groups. (D) Number of proteins identified per group is shown as means ± standard deviation (SD) (*n* = 3). (E) Cumulative protein abundance distribution. (F) Principal component analysis (PCA) of global proteomes. (G) Hierarchical clustering heatmap of quantified proteins. ND: normal diet; HFD: high-fat diet; HFDL: HFD + liraglutide (Lira); LC-MS/MS: liquid chromatography-tandem mass spectrometry; CV: coefficients of variation; FC: fold change; PC: principal component.

Label-free quantitative proteomics was used to analyze the global liver proteome of ND, HFD, and HFD + Lira (HFDL) mice (Fig. 2A). Most peptides ranged from 7 to 20 amino acids, consistent with expected enzymatic and mass spectrometric fragmentation patterns, meeting quality control standards (Fig. 2B). Moreover, the median abundance distribution of the quantified proteins was essentially located at the same level, indicating high-quality data suitable for further analysis (Fig. 2C). We filtered and retained proteins with at least one valid intensity value in any group for further analysis to ensure data quality. A total of 4,636 proteins were identified, with 1,799 proteins remaining after data filtering and preprocessing (Fig. 2D and Table S3). The quantifiable proteome covered a wide intensity range, spanning five orders of magnitude, indicating robust data coverage (Fig. 2E). Principal component analysis (PCA) revealed a distinct separation between ND and HFD groups. However, the HFDL group clustered closer to the ND group, suggesting that Lira treatment partially normalized the proteomic profile (Fig. 2F). Cluster and correlation analyses further supported these results, illustrating a high within-group correlation and a stronger correlation between ND and HFDL groups compared to HFD alone (Figs. 2G and S2). These findings indicate that Lira effectively corrects the liver proteome disrupted by a HFD.

**Fig. 3:**
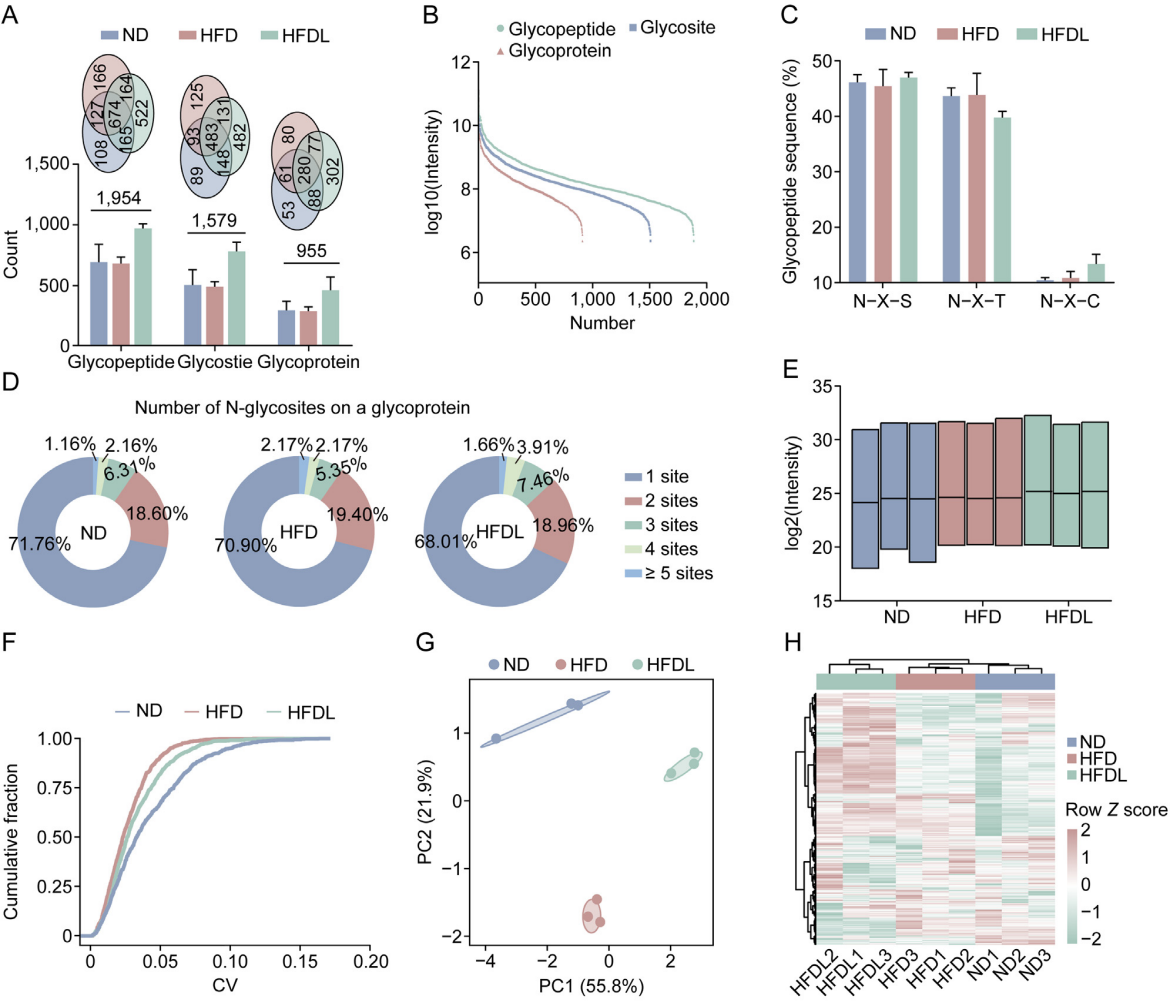
Overview of mouse liver glycoproteomics results. (A) The number of identified N-glycopeptides, N-glycosylation sites (N-glycosites), and N-glycoproteins is shown as means ± standard deviation (SD) (*n* = 3). (B) Cumulative abundance distribution of N-glycopeptides, N-glycosites, and N-glycoproteins. (C) Distribution of N−X−T/S/C (X ≠ proline) tripeptide sequences in identified N-glycopeptides is shown as means ± SD (*n* = 3). (D) Proportion of proteins containing varying numbers of N-glycosites. (E, F) Quality control analysis of the global liver glycoproteome: intensity distribution of N-glycosites (E) and coefficient of variation (CV) cumulative curve (F) for the indicated groups (*n* = 3 per group). (G) Principal component analysis (PCA) of global glycoproteomics. (H) Hierarchical clustering heatmap of quantified N-glycosites. ND: normal diet; HFD: high-fat diet; HFDL: HFD + liraglutide (Lira); PC: principal component.

In the glycoproteome analysis, 1,954 N-glycopeptides from 955 glycoproteins, containing 1,579 distinct N-glycosites, were identified across the ND, HFD, and HFDL groups (Table S4). The Venn diagram revealed that the HFDL group had the highest number of specific glycopeptides, glycoproteins, and N-glycosites (Fig. 3A). The intensity distribution of the N-glycosites, glycopeptides, and glycoproteins also spanned five orders of magnitude, reflecting robust data coverage (Fig. 3B). Most N-glycopeptides with N−X–S motifs being slightly more common than N−X−T motifs. Contrarily, N−X–C motifs accounted for about 10% (Fig. 3C). Distribution analysis exhibited that most glycoproteins (70%) had a single N-glycosite, with the HFDL group exhibiting a higher proportion of proteins with 2–4 N-glycosites, compared to ND and HFD groups (Fig. 3D). Motif analysis displayed only minor differences between the groups (Fig. S3). Similar to proteomics, we filtered N-glycosites, retaining those valid in over 50% of the samples in at least one group for analysis. After Lira treatment, 895 N-glycosites were retained for quantitative analysis (Table S4). Similar intensity distributions and low coefficient of variation (CV) (CV < 0.1) demonstrated high data quality (Figs. 3E and F). PCA analysis exhibited that the HFDL group clustered closer to the ND group, similar to the proteome results, although correlation analysis suggested that the HFDL group was not highly correlated with the ND group (Figs. 3G, 3H, and S4). These findings indicate that regulating protein glycosylation by Lira contributes to its therapeutic efficacy in ameliorating MASLD.

### 3.3 Lira modulated specific metabolic homeostasis by reducing proteomic differences and enhancing glycoproteomic variability

**Fig. 4:**
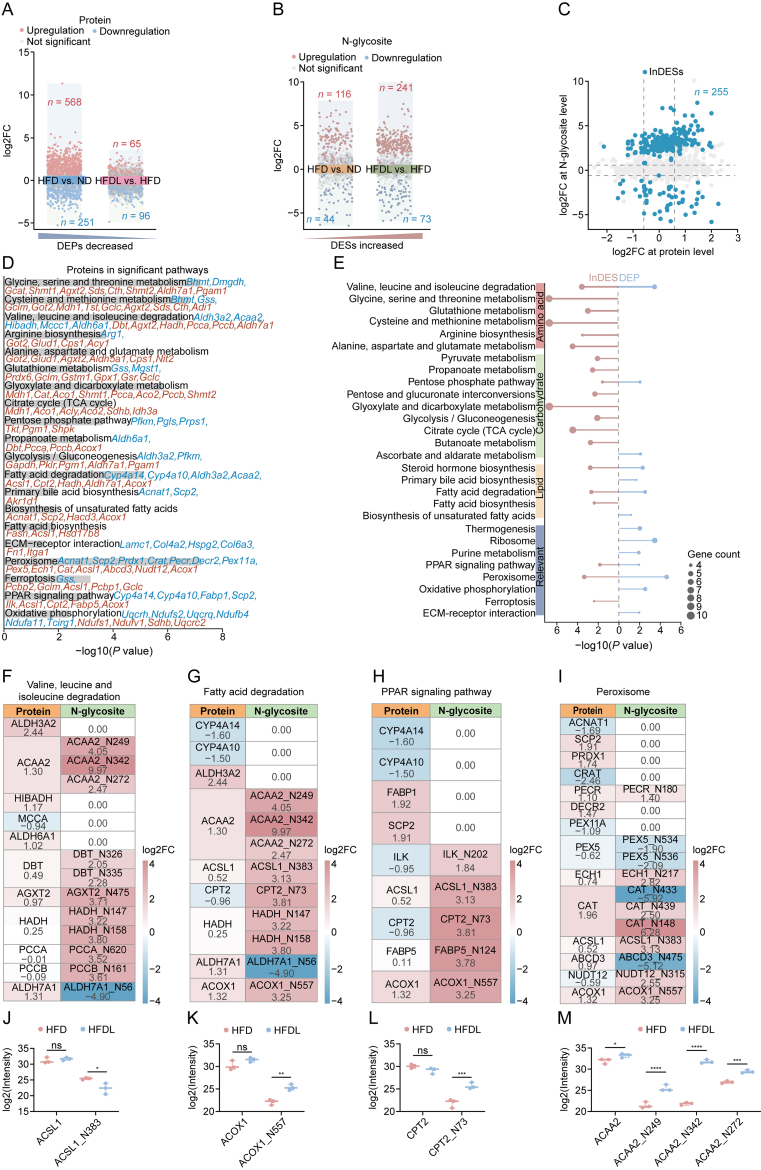
Comprehensive analysis of differentially expressed proteins (DEPs) and N-glycosylation sites (N-glycosites) (DESs) after liraglutide (Lira) treatment. (A) Volcano plots comparing DEPs across groups. (B) Volcano plots comparing DESs across groups. (C) Identification of independent differentially expressed N-glycosites (InDESs) with *P* < 0.05. (D) Kyoto Encyclopedia of Genes and Genomes (KEGG) pathway enrichment analysis on an integrative dataset of DEPs and InDESs (*P* < 0.05). The blue indicates DEPs and the red indicate glycoproteins from InDESs. (E) Comparative KEGG pathway differences in DEPs and InDESs (*P* < 0.05). (F–I) Details of distinct metabolic pathways significantly affected by Lira (0 indicates not identified in the glycoproteome): valine, leucine, and isoleucine degradation (F), fatty acid degradation (G), peroxisome proliferators-activated receptor (PPAR) signaling pathway (H), and peroxisome (I). (J–M) Intensity plot of specific proteins and their N-glycosites: acyl-CoA synthetase long-chain family member 1 (ACSL1) (J), acyl-CoA oxidase 1 (ACOX1) (K), carnitine palmitoyl transferase 2 (CPT2) (L), and acetyl-CoA acyltransferase 2 (ACAA2) (M). Data is presented as min to max (*n* = 3 per group). *P* values were calculated using Student's *t*-test. ^∗^*P* < 0.05, ^∗∗^*P* < 0.01, ^∗∗∗^*P* < 0.001, and ^∗∗∗∗^*P* < 0.0001. ns: not significant. FC: fold change; HFD: high-fat diet; ND: normal diet; HFDL: HFD + Lira; TCA: tricarboxylic acid cycle; ECM: extracellular matrix; ALDH3A2: aldehyde dehydrogenase family 3 member A2; HIBADH: 3-hydroxyisobutyrate dehydrogenase, mitochondrial; MCCA: methylcrotonoyl-CoA carboxylase subunit alpha; ALDH6A1: methylmalonate-semialdehyde/malonate-semialdehyde dehydrogenase, mitochondrial; DBT: dihydrolipoamide branched chain transacylase E2; AGXT2: alanine-glyoxylate aminotransferase 2; HADH: hydroxyacyl-CoA dehydrogenase; PCCA: propionyl-CoA carboxylase subunit alpha; CYP4A14: cytochrome P450 family 4 subfamily a polypeptide 14; FABP1: fatty acid-binding protein, liver; SCP2: sterol carrier protein 2; ILK: integrin linked kinase; PRDX1: peroxiredoxin 1; CRAT: carnitine *O*-acetyltransferase; PECR: peroxisomal *trans*-2-enoyl-CoA reductase; DECR2: 2,4-dienoyl-CoA reductase 2; PEX11A: peroxisomal membrane protein 11A; PEX5: peroxisomal targeting signal 1 receptor; ECH1: delta(3,5)-delta(2,4)-dienoyl-CoA isomerase, mitochondrial; ABCD3: adenosine triphosphate (ATP)-binding cassette sub-family D member 3; NUDT12: NAD-capped RNA hydrolase NUDT12.

A *t*-test-based abundance difference analysis was used to identify differentially expressed proteins (DEPs) and differentially expressed N-glycosites (DESs) following Lira treatment. In proteome analysis, 819 DEPs were identified in the HFD group compared to the ND group (|fold change (FC)| > 1.5, *P* < 0.05), with 568 proteins upregulated and 251 downregulated. In contrast, the HFDL group demonstrated 161 DEPs compared to the HFD group, with 65 upregulated and 96 downregulated proteins, as depicted in the volcano plot (Fig. 4A and Table S5). In glycoproteome analysis, 160 DESs were identified between HFD and ND groups, with 116 upregulated and 44 downregulated. Remarkably, Lira treatment resulted in 314 DESs compared to the HFD group, with a substantial increase in upregulated sites (241) compared to downregulated sites (73) (Fig. 4B and Table S5).

To further evaluate whether the differences in *N*-glycopeptide abundance were due to altered protein expression or changes in N-glycosites occupancy, a comparative analysis was conducted between DEPs and DESs. This integrated analysis of proteomic and glycoproteomic data identified 255 differential sites, termed independent DESs (InDESs), which were independent of overall protein level changes and were derived from 218 glycoproteins (Fig. 4C and Table S5). These results suggested that although Lira induced obvious changes in protein expression levels, it had a more pronounced effect on glycosylation. This indicated the importance of glycosylation regulation in Lira therapeutic regulation and its unique role in molecular regulation for MASLD therapy.

GO and KEGG pathway enrichment analyses were performed on an integrative dataset of DEPs and InDESs to reveal the mechanisms by which Lira ameliorates MASLD. KEGG analysis, exhibiting DEPs in blue and glycoproteins from InDESs in red, revealed that Lira primarily influenced amino acid, carbohydrate, and lipid metabolism, as well as peroxisome, peroxisome proliferator-activated receptor (PPAR) signaling pathway, and oxidative phosphorylation pathways. Additionally, extracellular matrix (ECM)-receptor interaction and ferroptosis pathways were significantly enriched, suggesting antifibrotic and antioxidant effects (Fig. 4D and Table S6). Similarly, GO enrichment identified key roles in nucleotide, fatty acid, and amino acid metabolism (Figs. S5−S7 and Table S6). Notably, over half of the pathways were predominantly enriched in glycoproteins. The lollipop plot indicated that Lira regulation of InDESs focused on amino acid and carbohydrate metabolism, while DEPs primarily affected lipid metabolism and fibrosis-related pathways (Fig. 4E and Table S6).

As demonstrated in Fig. 4E, glycoproteomic analysis specifically revealed substantial enrichment in pathways related to glycine, serine, threonine, cysteine, and methionine metabolism, as well as alanine, aspartate, glutamate metabolism, arginine biosynthesis, and glutathione metabolism. Lira significantly influenced the degradation pathways of branched-chain amino acids (BCAAs) such as valine, leucine, and isoleucine, with notably different FC observed between the proteome and glycoproteome at the protein and N-glycosite levels (Fig. 4F). Glutathione metabolism and ferroptosis were notably enriched in the glycoproteome. Additionally, glycoproteomic data demonstrated broad effects on carbohydrate metabolism, including the citrate cycle, propanoate metabolism, pentose phosphate pathway, and glycan synthesis, underscoring the role of Lira in energy production and glycosylation, which are crucial for liver function and MASLD improvement. In contrast, the proteomic analysis highlighted the significant effects of Lira on fatty acid degradation, steroid hormone biosynthesis, bile acid biosynthesis, oxidative phosphorylation, and the PPAR signaling pathway, indicating enhanced fatty acid oxidation. The enrichment of ECM-receptor interaction in the proteome suggested a role for Lira in mitigating liver fibrosis associated with MASLD [22].

The distinct differences between glycosylation and protein-level changes are further clearly detailed in Figs. 4G−I. For instance, for acyl-CoA synthetase long-chain family member 1 (ACSL1), ACAA2, acyl-CoA oxidase 1 (ACOX1), and carnitine palmitoyl transferase 2 (CPT2), which appeared in at least two pathways, it was found that N-glycosites exhibited greater variability under Lira treatment compared to proteins (Figs. 4J−M). These results revealed that glycoproteomics provides deeper molecular insights, complementing proteomics and revealing distinct and unique regulatory roles in understanding complex biological pathways.

### 3.4 Identification and validation of signature proteins and glycoproteins modulated by Lira

**Fig. 5:**
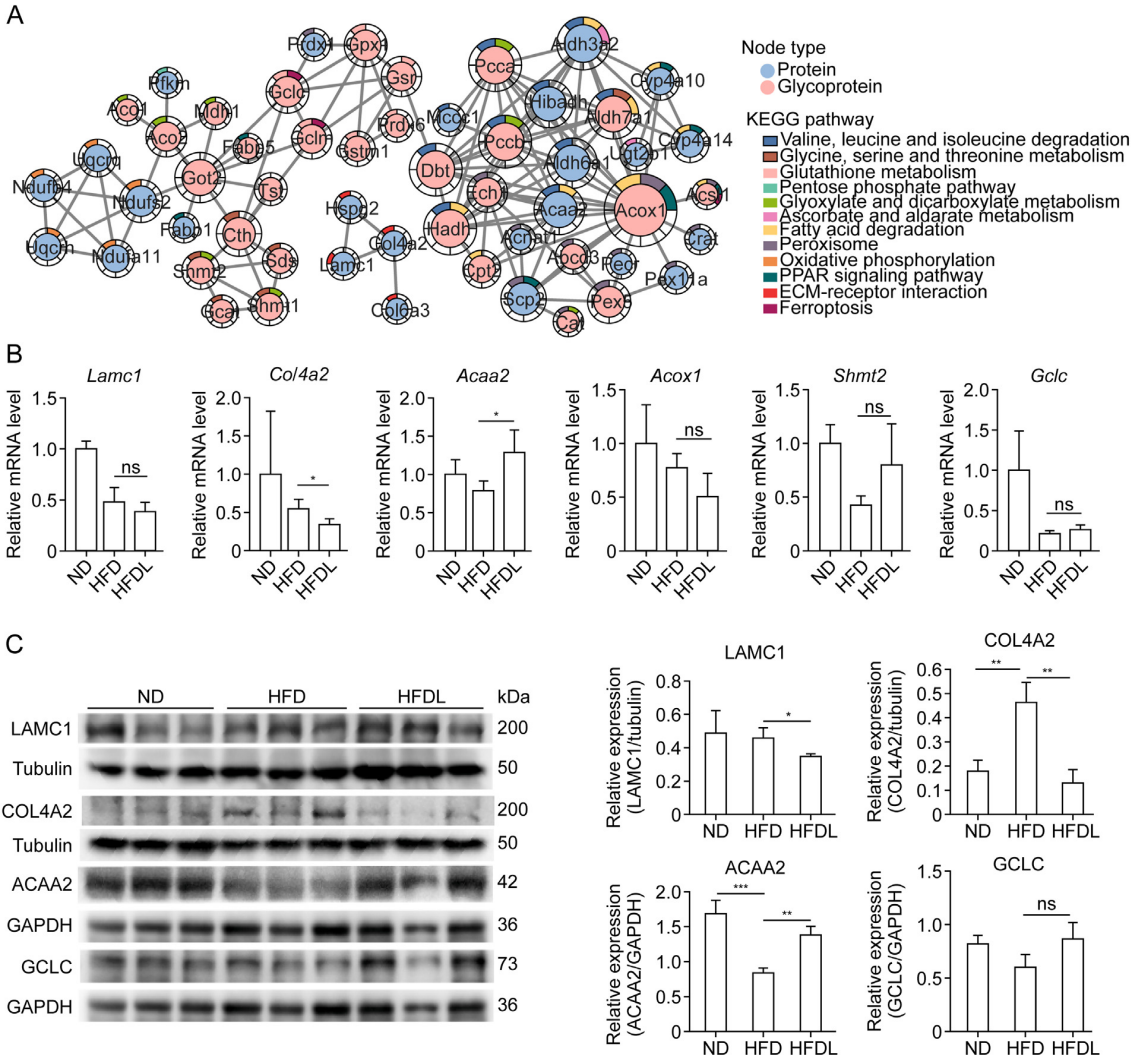
Identification and validation of potential signature molecules affected by liraglutide (Lira). (A) Protein-protein interaction (PPI) analysis of differential proteins and glycoproteins in major pathways. Node size reflects degree value, with red representing glycoproteins and blue representing proteins. Color-coded segments show pathway associations. (B) Messenger RNA (mRNA) expression of *Acaa2*, *Lamc1*, and *Col4a2* (for proteins), and *Acox1*, *Gclc*, and *Shmt2* (for glycoproteins). (C) Protein expression of laminin subunit gamma-1 (LAMC1), collagen type IV alpha-2 chain (COL4A2), and acetyl-CoA acyltransferase 2 (ACAA2) (for proteins), and glutamate-cysteine ligase catalytic subunit (GCLC) (for glycoproteins). Data is presented as mean ± standard deviation (SD) (*n* = 3 per group). *P* values were calculated using Student's *t*-test or one-way analysis of variance (ANOVA) with Tukey's comparisons. ^∗^*P* < 0.05, ^∗∗^*P* < 0.01, and ^∗∗∗^*P* < 0.001. ns: not significant. PPAR: peroxisome proliferators-activated receptor; ECM: extracellular matrix; ND: normal diet; HFD: high-fat diet; HFDL: HFD + Lira; GAPDH: glyceraldehyde-3-phosphate dehydrogenase.

The effects of Lira on MASLD were further explored through PPI analysis of the DEPs and InDESs (Fig. 5A and Table S7). Proteins or glycoproteins involved in two or more metabolic pathways were identified as key nodal molecules, indicating that these are the potential primary targets regulated by Lira treatment. In the proteome, proteins involved in multiple pathways, such as aldehyde dehydrogenase 3 family member A2 (ALDH3A2), ACAA2, cytochrome P450 family 4 subfamily A member 10 (CYP4A10), and sterol carrier protein 2 (SCP2), were identified as central to Lira action, particularly in fatty acid degradation and antifibrotic effects, with LAMC1 and COL4A2 also being significant. In the glycoproteome, GCLC, glutamate-cysteine ligase modifier subunit (GCLM), serine hydroxy methyl transferase 2 (SHMT2), and ACOX1 were highlighted, involving 14 InDESs, suggesting that Lira strongly influences glycosylation. The specific changes observed in these key molecules are listed in Table 1. These findings suggest that the therapeutic effects of Lira on MASLD are mediated by regulating these critical proteins, specifically in glycosylation pathways.

**Table 1:** Signature molecules in liraglutide (Lira) therapy for metabolic dysfunction-associated steatotic liver disease (MASLD).

Gene	Protein ID	N-glycosite	log2FC (HFDL/HFD)	−log10(*P* value)	Degree	Type
*Aldh3a2*	P47740	–	2.44	1.34	10	DEP
*Acaa2*	Q8BWT1	–	1.30	1.35	9	DEP
*Cyp4a10*	O88833	–	−1.50	1.49	4	DEP
*Cyp4a14*	O35728	–	−1.60	1.32	4	DEP
*Scp2*	P32020	–	1.91	1.64	7	DEP
*Lamc1*	P02468	–	−2.64	1.51	2	DEP
*Col4a2*	P08122	–	−1.18	1.80	3	DEP
*Acox1*	Q9R0H0	ACOX1_N557	3.25	2.47	18	InDES
*Acsl1*	P41216	ACSL1_N383	3.13	1.43	1	InDES
*Aldh7a1*	Q9DBF1	ALDH7A1_N56	−4.90	3.48	9	InDES
*Cat*	P24270	CAT_N433	−5.92	2.82	1	InDES
		CAT_N439	2.50	2.78		InDES
		CAT_N148	6.28	1.85		InDES
*Gclc*	P97494	GCLC_N598	3.42	1.53	6	InDES
*Gclm*	O09172	GCLM_N101	1.70	1.45	5	InDES
*Hadh*	Q61425	HADH_N147	3.22	2.39	10	InDES
		HADH_N158	3.80	2.37		InDES
*Shmt1*	P50431	SHMT1_N183	4.00	1.94	4	InDES
*Shmt2*	Q9CZN7	SHMT2_N78	2.78	1.74	4	InDES
*Pcca*	Q91ZA3	PCCA_N620	3.52	2.54	10	InDES
*Pccb*	Q99MN9	PCCB_N161	3.61	2.91	10	InDES

−: no data. N-glycosite: N-glycosylation site; FC: fold change; HFDL: high-fat diet (HFD) + liraglutide (Lira); DEP: differentially expressed protein; ACOX1: acyl-CoA oxidase 1; InDES: independent differentially expressed N-glycosites; ACSL1: acyl-CoA synthetase long-chain family member 1; ALDH7A1: aldehyde dehydrogenase 7 family member A1; CAT: catalase; GCLC: glutamate-cysteine ligase catalytic subunit; GCLM: glutamate-cysteine ligase modifier subunit; HADH: hydroxyacyl-coenzyme A dehydrogenase; SHMT1: serine hydroxymethyl transferase 1; PCCA: propionyl-coenzyme A carboxylase alpha subunit.

Six signature molecules were selected for gene expression analysis, based on log2FC and degree values, which are involved in pathways such as amino acid and fatty acid metabolism, and antioxidant and antifibrotic mechanisms. Specifically, ACAA2, LAMC1, and COL4A2 were identified in DEPs, while ACOX1, GCLC, and SHMT2 were recognized as glycoproteins linked to InDESs. Lira treatment reduced the messenger RNA (mRNA) expression of *Lamc1*, and *Col4a2*, and increased *Acaa2* expression compared to the HFD group (Fig. 5B). This aligned with proteomic findings, affirming data accuracy. Notably, Lira significantly regulated ECM-receptor interactions, particularly by decreasing LAMC1 and COL4A2 protein levels, suggesting a potential mechanism for ameliorating hepatic fibrosis (Fig. 5C). Furthermore, Lira significantly increased the protein level of ACAA2 (Fig. 5C), and these results were consistent with the analysis of proteomics. Glycoprotein evidence demonstrated that Lira decreased *Acox1* mRNA expression while increasing *Gclc* and *Shmt2*. However, these trends were non-significant (Fig. 5B). Similarly, GCLC did not change significantly at the protein level (Fig. 5C). This suggests that Lira effect on glycosylation may be independent of changes in the overall protein expression.

### 3.5 Hydrogel-based drug delivery extended the dosing interval of Lira while maintaining its therapeutic efficacy

Given that clinical Lira treatment requires several months for sustained efficacy, we extended the treatment duration to assess long-term effects. However, with a half-life of approximately 12 h, daily injections are necessary to maintain therapeutic levels, compromising patient compliance [23,24]. Injectable hydrogels offer a promising solution by enabling sustained drug release, reducing injection frequency, and enhancing adherence [[25], [26], [27]]. Fibrin (Fib), a biodegradable natural protein, provides a stable scaffold for liraglutide encapsulation and controlled release without altering its structure or function [[28], [29], [30], [31], [32]]. This approach would improve therapeutic efficiency while minimizing the burden of frequent administration.

**Fig. 6:**
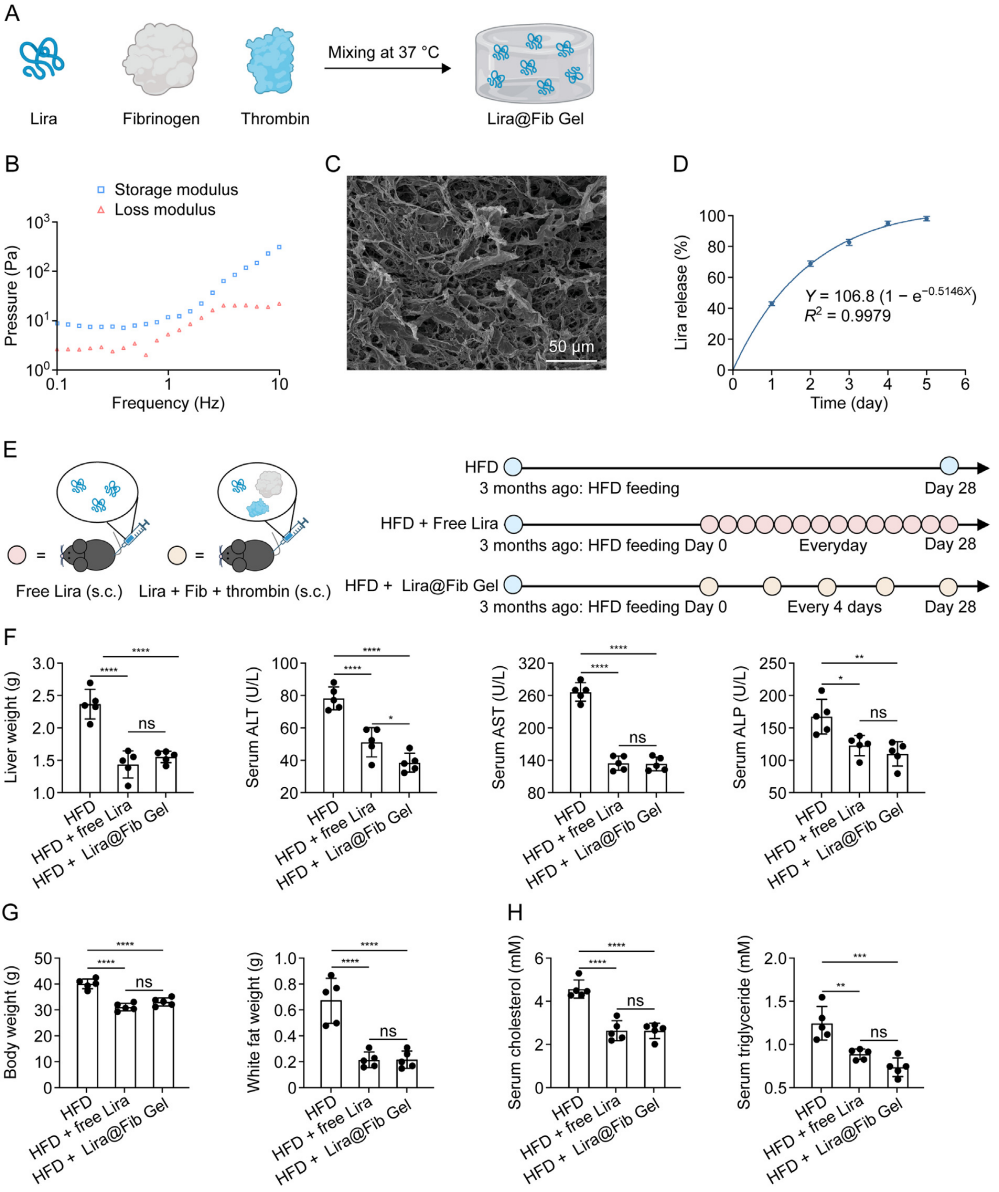
Hydrogel-based liraglutide (Lira) delivery (Lira@fibrin (Fib) Gel) reduced dosing frequency without compromising therapeutic efficacy in long-term metabolic dysfunction-associated steatotic liver disease (MASLD) treatment in C57/BL6 mice. (A) Schematic illustration of Lira@Fib Gel preparation. (B–D) Characterization of Lira@Fib Gel: rheological properties (B), microstructure (C), and drug release profile (D). (E) Treatment schedule. (F–H) Therapeutic effects of free Lira and Lira@Fib Gel: protection against liver injury (F), inhibition of epididymal white fat accumulation and body weight gain (G), and modulation of lipid metabolism (H). Data is presented as mean ± standard deviation (SD) (*n* = 5 per group). Statistical significance was calculated using one-way analysis of variance (ANOVA) with Tukey's multiple comparisons. ^∗^*P* < 0.05, ^∗∗^*P* < 0.01, ^∗∗∗^*P* < 0.001, and ^∗∗∗∗^*P* < 0.0001. ns: not significant. HFD: high-fat diet.

The Lira@Fib Gel was prepared by mixing Lira, fibrinogen, and thrombin (Fig. 6A). The Lira-loaded Fib hydrogel (Lira@Fib Gel) was prepared by mixing Lira, fibrinogen, and thrombin (Fig. 6A). Lira was first dissolved in a fibrinogen solution, after which thrombin was added. Following thorough mixing, the solution was immediately injected subcutaneously into mice. At body temperature, thrombin cleaves fibrinogen into Fib monomers that subsequently crosslink to form a gel-like network, effectively encapsulating Lira. Rheological measurements confirmed that after gelation, the storage modulus of Lira@Fib Gel exceeded the loss modulus, indicating the formation of a stable, non-flowing solid (Fig. 6B). Scanning electron microscopy (SEM) of the hydrogel's cross-sectional microstructure revealed a uniform porous network (Fig. 6C). As body fluids interact with the gel and the matrix gradually degrades, Lira is released in its free form in a sustained manner and is subsequently absorbed to exert its therapeutic effects. The release profile of Lira from Lira@Fib Gel closely followed first-order kinetics (*R*^2^ = 0.9979), with no evident burst release (Fig. 6D). After four days, cumulative drug release exceeded 80%, indicating a relatively complete release from the hydrogel formulation. To confirm that the Fib hydrogel functions solely as a drug reservoir, HFD-fed mice treated with drug-free Fib hydrogel (Fib Gel) showed hepatic pathology identical to the untreated group (Fig. S8), indicating no independent effect on MASLD treatment.

Based on the drug release behavior of Lira@Fib Gel (Fig. 6D), a dosing interval of four days was adopted for *in vivo* applications (Fig. 6E). Over a 28-day therapeutic period, both Lira@Fib Gel and daily free Lira injections effectively alleviated liver injury, reduced fat accumulation, and promoted lipid metabolism (Figs. 6F−H and S9). These results demonstrate that Lira@Fib Gel achieves therapeutic outcomes comparable to the standard clinical treatment regimen while reducing the frequency of subcutaneous injections by 75%. In clinical practice, this approach could significantly reduce the discomfort associated with frequent injections, thereby minimizing the risks of local skin irritation and infection and improving patient compliance. Furthermore, after 28 days of Lira@Fib Gel treatment, serum levels of TP, ALB, LDH, CKMB, amylase, creatinine, and UA did not differ significantly from those in the untreated group (Fig. S10), indicating that Lira@Fib Gel exhibits excellent biosafety without causing damage to the heart, pancreas, or kidneys.

## 4 Discussion

Lira, a GLP-1 receptor agonist approved by the FDA for diabetes and obesity, can improve liver health by reducing hepatic steatosis and inflammation. However, a systematic and comprehensive analysis of its molecular regulatory networks, targets, and effects, which are essential for expanding its therapeutic use and guiding MASLD treatment development, is still lacking. We observed that Lira treatment rapidly reduced hepatic steatosis and systemic fat within one week in HFD-induced MASLD C57 mice (Fig. 1), indicating its potential as an adjunct to lifestyle modifications for accelerating weight loss and lipid reduction in MASLD patients. The proteome and glycoproteome profiling identified 4,636 proteins and 1,954 *N*-glycopeptides from 955 glycoproteins, covering 1,579 distinct N-glycosites, demonstrating thorough and reliable data (Fig. 2, Fig. 3A). Notably, Lira treatment resulted in 161 DEPs and 255 InDESs, with a stronger impact on glycosylation than protein expression, underscoring the crucial role of glycosylation regulation in its therapeutic effects on MASLD (Figs. 4A and C).

Our proteomics results revealed that Lira treatment regulated critical pathways involved in MASLD improvement, including amino acid, lipid, and carbohydrate metabolism, and ECM remodeling. These findings align with previous multi-omics studies on the diet-induced obese (DIO)-MASH model, which identified similar key features, such as impaired lipid metabolism and ECM alterations [33]. The fatty acid degradation pathway, particularly β-oxidation, was a primary mechanism by which Lira reduces hepatic steatosis [34]. Impaired fatty acid oxidation, a hallmark of MASH, is characterized by reduced long-chain fatty acid oxidation and β-HAD activity [35,36]. Lira treatment reduced oxidative stress and lipid accumulation, as demonstrated by the increased expression of ACAA2, a signature molecule that enhancs β-oxidation (Fig. 4, Fig. 5C). Additionally, CYP4A14 and CYP4A10 downregulation in the fatty acid degradation pathway (Fig. 4G) further supported the Lira role in promoting fatty acid oxidation and reducing hepatic fat accumulation [[37], [38], [39]]. Activation of the peroxisome pathway implies that Lira promotes peroxisomal β-oxidation via PPARα, improves hepatic function, and supports its therapeutic role in MASLD [40].

The differential expression analysis of N-glycosites revealed key regulatory mechanisms of Lira beyond protein expression. For instance, ACOX1 and CPT2, which are crucial for fatty acid oxidation [10,41], were significantly upregulated at the N-glycosites level, specifically at ACOX1_N557 for ACOX1 and CPT2_N73 for CPT2, demonstrating that Lira enhances glycosylation occupancy to promote these processes, which is consistent with previous reports on mitochondrial biogenesis and fatty acid oxidation [42] (Figs. 4K and L). Fatty acid β-oxidation increases H_2_O_2_ production, which can induce oxidative stress. Catalase (CAT) decomposes H_2_O_2_, maintaining redox balance and protecting cells [43]. Lortz et al. [44] identified N-glycosites (N244 and N439) in CAT, with mutations at N244 impairing activity. Our study revealed Lira-induced upregulation of CAT_N439 glycosylation, potentially enhancing CAT activity, promoting β-oxidation, and reducing oxidative stress in the liver.

Additionally, fibrosis-related pathways were significantly enriched in the proteomics analysis, particularly the ECM-receptor interaction pathway. In the HFD group, DEPs, including COL4A2, COL6A3, and LAMC1 were upregulated, while Lira treatment led to their significant downregulation (Table S5). Notably, COL4A2 and LAMC1 were closely associated with ECM-receptor interactions, suggesting that Lira mitigates liver fibrosis by inhibiting these pathways. Our findings align with previous multi-omics studies exhibiting ECM-receptor interaction involvement in MASLD [45], with Lira downregulating LAMC1 (Fig. 5C) and improving fibrosis despite prior inconsistencies [46]. Notably, LAMC1, an ECM glycoprotein, and COL4A2, a type IV collagen, were identified in the proteome but may have been partially lost during protein extraction due to their membrane-bound and matrix-associated nature [47,48].

Remarkably, glycoproteomic analysis unveiled unique insights into the modulation of specific metabolic pathways by Lira, particularly highlighting its distinct impact on glycosylation, amino acid and carbohydrate metabolism, and ferroptosis, which were not as evident in the proteomic analysis (Fig. 4E). Amino acid metabolism is crucial for glycosylation, as essential amino acids, such as serine, threonine, and asparagine, serve as attachment sites for N-glycans and O-glycans. Lira-modulated pathways, including alanine, aspartate, glutamate, and glutathione metabolism, directly impact glycosylation by sustaining glycosyl donors and redox balance. Cysteine and methionine metabolism disruptions may impair glycosylation and further contribute to MASLD progression. Lira specifically promotes BCAA degradation, which is often elevated in hepatic steatosis and is associated with obesity, insulin resistance, and T2DM. Lira may reduce lipotoxicity and promote fatty acid oxidation by enhancing BCAA degradation and boosting glycine metabolism, thereby ameliorating MASLD [49,50].

Moreover, Lira significantly upregulated ferroptosis-related proteins, including ACSL1, GCLM, and GCLC, indicating enhanced glutathione production, which protects cells from ferroptosis, a lipid peroxidation-related cell death process associated with liver disease [[51], [52], [53]]. Furthermore, GCLM and GCLC, which are crucial proteins in glutathione biosynthesis, were uniquely upregulated at the N-glycosites level, specifically at GCLC_N598 and GCLM_N101 (Fig. 4D and Table 1). However, impaired glutathione metabolism is well-documented in patients with MASH [54]. Our study is the first to exhibit that Lira enhances the glycosylation of these proteins, potentially promoting glutathione synthesis and reducing oxidative stress [55,56]. Lira also significantly regulated carbohydrate metabolism through pathways including the pentose phosphate, glyoxylate, and dicarboxylate metabolism and the TCA cycle, ensuring glycan precursor availability and cellular energy balance, thereby supporting protein stability and mitigating MASLD progression (Fig. 4E).

We further analyzed glycosyltransferases (GTs), enzymes that transfer sugar residues to receptor molecules, and downloaded 50 GTs (Mus musculus C57BL/6J) from the Genome Taxonomy Database (GTDB) [57,58]. Integrating these with DEPs revealed significant upregulation of UGT1A6 and UGT3A2 after Lira treatment (downregulated in the HFD group) (Fig. S11 and Table S8). Uridine diphosphate (UDP)-glucuronosyltransferases (UGTs), localized in the endoplasmic reticulum, catalyze glycosyl transfer using UDP-glucuronic acid. Abnormal UGT expression in MASLD livers has been reported, with downregulation of UGT1A9 and UGT1A6 observed in MASH [57,59]. Consistently, our findings suggest that Lira upregulates UGT1A6 and UGT3A2, potentially promoting glycosylation and contributing to its therapeutic effect. Additionally, heavily glycosylated transferrin, known as MASLD biomarker [56], illustrated significantly reduced glycosylation after Lira treatment (Table S5), indicating Lira may improve MASLD via GTs regulation.

Injectable hydrogels are a major advancement in drug delivery, offering controlled and sustained drug release by forming a localized drug depot after administration, which can reduce the dosing frequency of free drugs. Given the rapid clearance rate of Lira, which requires daily injections, long-term MASLD treatment may lead to severe adverse effects. To address this limitation, we developed Lira@Fib Gel, a long-acting injectable hydrogel formulation. Our study demonstrated that the Lira@Fib Gel formulation effectively reduces dosing frequency while maintaining therapeutic efficacy comparable to daily injections (Fig. 6), providing a promising strategy for clinical translation. The formulation exhibited excellent biosafety, with no detectable organ damage (Fig. S10). As a biologically inert drug depot, the Fib hydrogel is expected to influence only the pharmacokinetics of Lira without altering its associated biomarkers. Nonetheless, future studies incorporating parallel proteomics and glycoproteomics analyses in Lira@Fib Gel-treated mice could further validate this assumption.

## 5 Conclusion

Herein, integrative proteomic and glycoproteomic analyses revealed that Lira exerted its therapeutic effects on MASLD primarily through significant changes in glycosylation, with more pronounced effects than on overall protein expression. Glycoproteomic analysis highlighted pathways related to amino acid metabolism, carbohydrate metabolism, and ferroptosis, emphasizing the Lira's role in glycosylation regulation. Conversely, proteomic analysis has focused on PPAR-regulated lipid metabolism and ECM-receptor interaction, reflecting its impact on lipid metabolism and fibrosis. Identifying signature molecules and N-glycosites provided potential therapeutic targets, and further development of a long-acting hydrogel formulation offered enhanced delivery and sustained efficacy, advancing chronic metabolic disease management. These findings presented a landscape of Lira regulatory networks and targets, offering critical insights into MASLD therapy and advancing clinical translation.

## CRediT authorship contribution statement

**Yuxuan Chen:** Writing – review & editing, Writing – original draft, Visualization, Validation, Investigation, Data curation. **Chendong Liu:** Writing – review & editing, Writing – original draft, Visualization, Validation, Investigation, Data curation. **Qian Yang:** Visualization, Investigation. **Jingtao Yang:** Visualization, Investigation. **He Zhang:** Investigation. **Yong Zhang:** Resources. **Yanruyu Feng:** Investigation. **Jiaqi Liu:** Investigation. **Lian Li:** Writing – review & editing, Supervision. **Dapeng Li:** Writing – review & editing, Supervision, Funding acquisition.

## Data availability

All raw data associated with this manuscript have been deposited to the ProteomeXchange Consortium via the PRoteomics IDEntification Database (PRIDE) partner repository with the dataset identifier PXD055468.

## Declaration of competing interest

The authors declare that there are no conflicts of interest.
