# Supplementary material for: Signatures of proteomics and glycoproteomics revealed liraglutide ameliorates MASLD by regulating specific metabolic homeostasis in mice
Source: J Pharm Anal. 2025 Mar 19;15(11):101273. doi: 10.1016/j.jpha.2025.101273 (PMC12688686; doi:10.1016/j.jpha.2025.101273)
Supplement: Multimedia component 1 [file mmc1.docx]

# Supporting Material

**Signatures of Proteomics and Glycoproteomics Revealed Liraglutide Ameliorates MASLD by Regulating Specific Metabolic Homeostasis in Mice**

**Table S1. Reagents for automatic biochemical analyzer.**

| **Biochemical Parameter** | **Catalog Number of Reagent** |
| --- | --- |
| Alkaline Phosphatase (ALP) | 03333701190 |
| Alanine Aminotransferase (ALT) | 20764957322 |
| Aspartate Aminotransferase (AST) | 20764949322 |
| Low-Density Lipoprotein (LDL) | 07005717190 |
| High-Density Lipoprotein (HDL) | 07528566190 |
| Triglycerides (TG) | 20767107322 |
| Total Protein (TP) | 03183734190 |
| Albumin (ALB) | 04469658190 |
| Lactate Dehydrogenase (LDH) | 03004732112 |
| Creatine Kinase-MB (CKMB) | 07190808190 |
| α-Amylase | 03183742122 |
| Urea | 04460715190 |

**Table S2. Primer sequences used for RT-PCR.**

| Species | Gene | Primer Sequence 5’-3’ |
| --- | --- | --- |
| Mouse | *Acaa2* | Forward: TCGATAGTGTCATCGTGGGC |
|  |  | Reverse: GAGCCTGTTGAGGGTAAGGG |
| Mouse | *Lamc1* | Forward: TTGGGTTTGGACCTGAAGGC |
|  |  | Reverse: CTTCACACTGGTCACAGCGA |
| Mouse | *Col4a2* | Forward: GAAACAGGAGCACCGGGATT |
|  |  | Reverse: TCTCCAGGCAAACCTCCAAG |
| Mouse | *Acox1* | Forward: TGGGCACGGCTATTCTCAC |
|  |  | Reverse: CCCGACTGAACCTGGTCATA |
| Mouse | *Gclc* | Forward: CACATCTACCACGCAGTCAAG |
|  |  | Reverse: ATCGCCTCCATTCAGTAACAAC |
| Mouse | *Shmt2* | Forward: AGACATGGCCCACATCAGTG |
|  |  | Reverse: CCTCGCAGTGTCTTGTGAGT |
| Mouse | *β-actin* | Forward: CATCCGTAAAGACCTCTATGCCAAC |
|  |  | Reverse: ATGGAGCCACCGATCCACA |

**Table S3: Proteome profiling of Mice liver. (XLSX)**

**Table S4: Glycoproteome profiling of Mice liver. (XLSX)**

**Table S5: Differential expression analysis of proteomes and glycoproteomes. (XLSX)**

**Table S6: Results of Gene Ontology (GO) and Kyoto Encyclopedia of Genes and Genomes (KEGG) analysis. (XLSX)**

**Table S7: Results of the Protein-protein interaction (PPI) analysis. (XLSX)**

**TableS8: Integrating identified proteins, differentially expressed proteins (DEPs) and glycosyltransferases (GTs) from the database. (XLSX)**


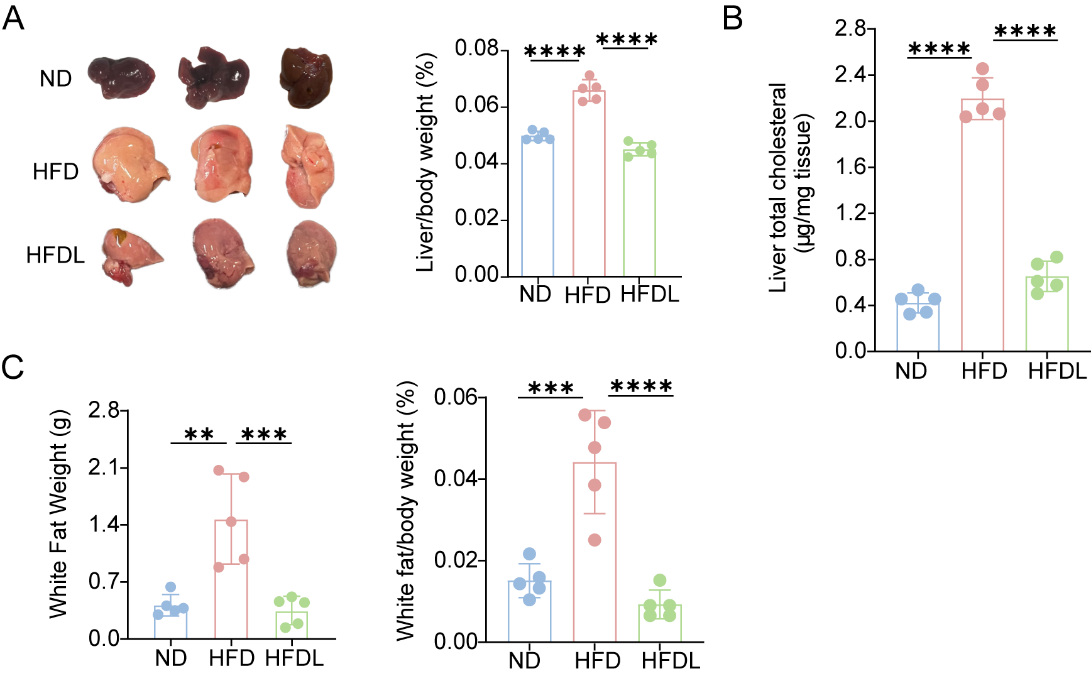


**Fig. S1.** Supplementary data for the therapeutic outcomes of short-term daily liraglutide administration presented in Figure 1. (A) Liver morphology and liver to body weight ratio at the endpoint. (B) Quantification of total cholesterol content in the liver. (C) Epididymal white fat weight and fat to body weight ratio at the endpoint. *N* = 5 animals per group, data presented as mean ± SD. Statistical significance was calculated using one-way ANOVA with Tukey’s multiple comparisons. ***P*  <  0.01, ****P*  <  0.001, *****P*  <  0.0001, ns: not significant.


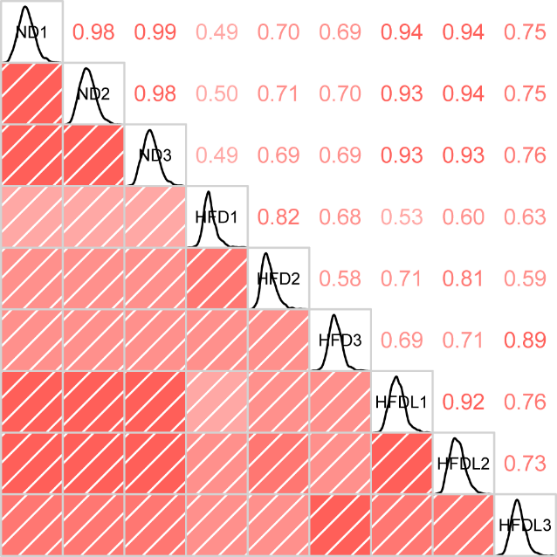


**Fig. S2.** Pearson correlation analysis of different groups of the liver proteome.


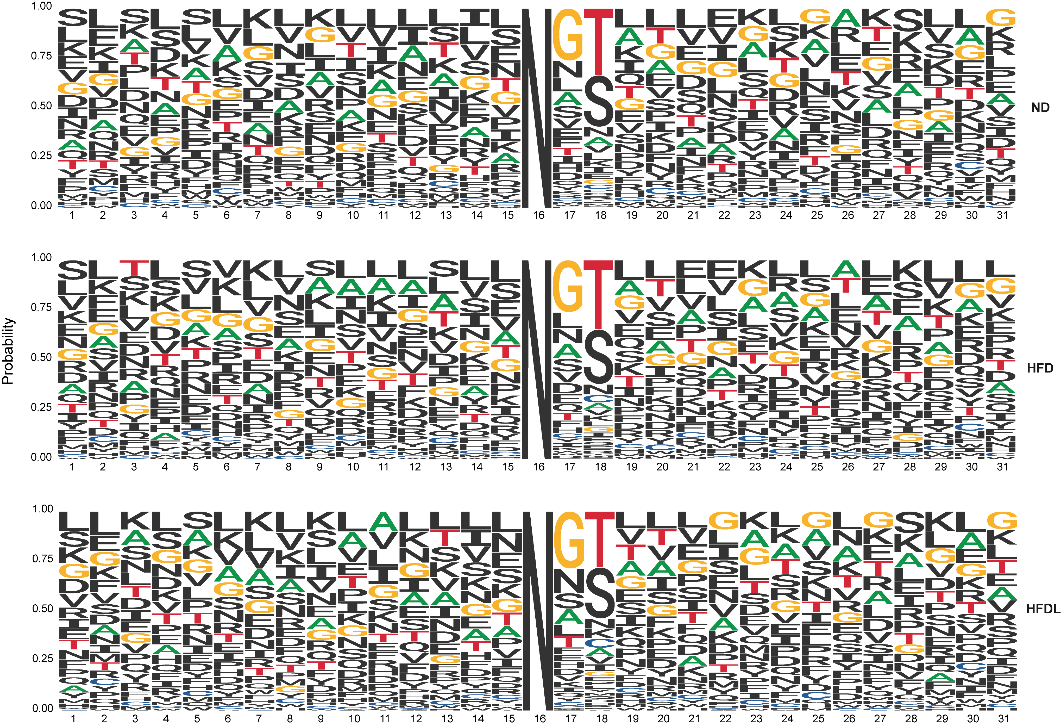


**Fig. S3.** Motif analysis between groups.


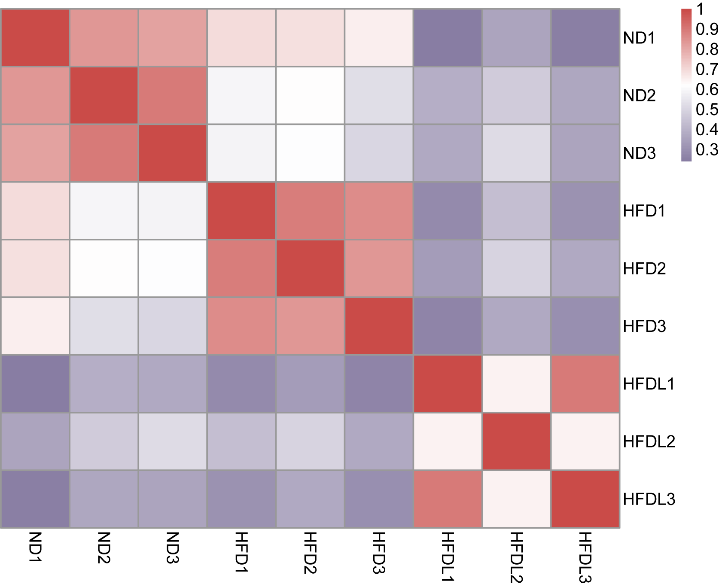


**Fig. S4.** Pearson correlation analysis of different groups of the liver glycoproteome.


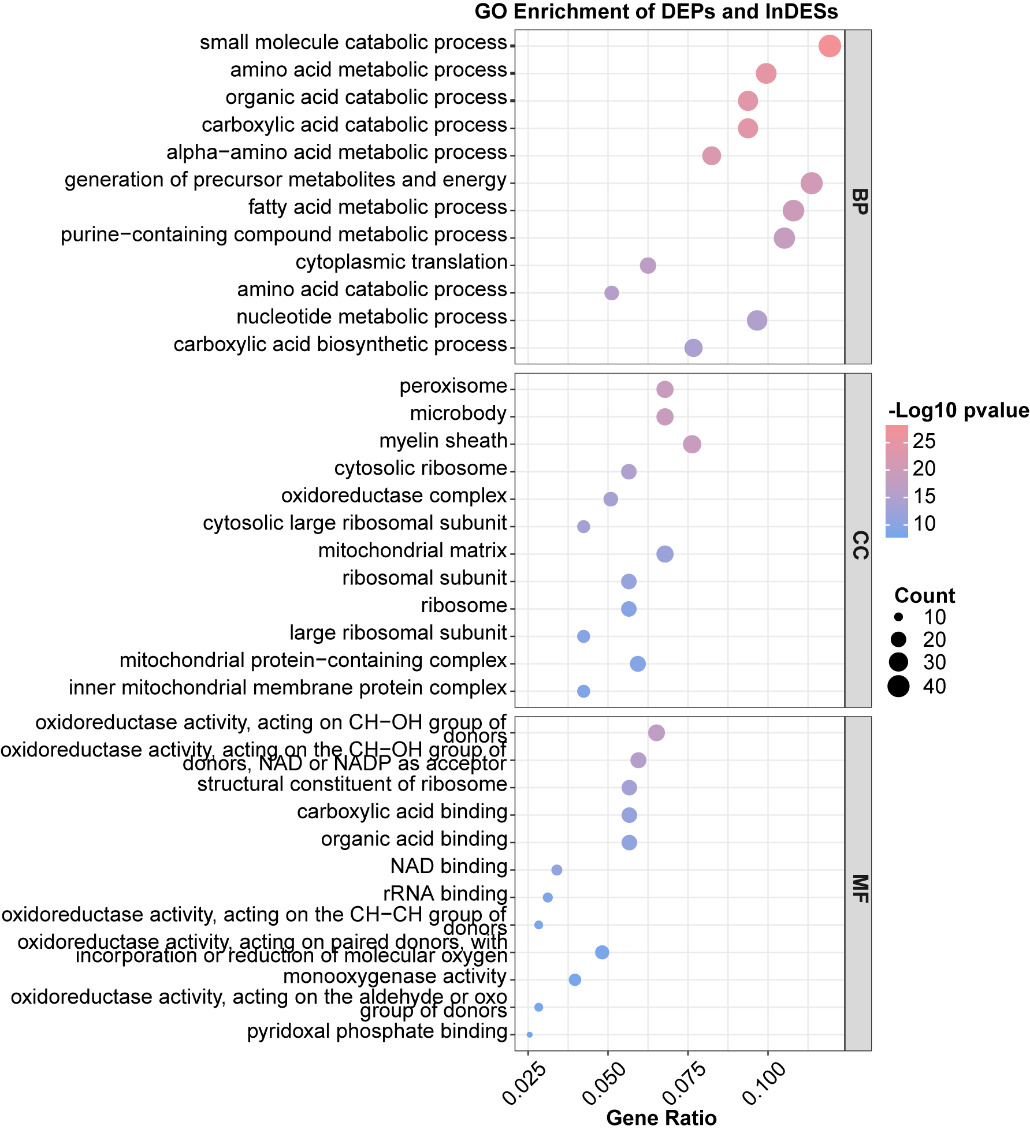


**Fig. S5.** Gene Ontology (GO) terms of differentially expressed proteins (DEPs) and independent differential expression N-glycosites (InDESs). BP: Biological Process, CC: Cellular Component, MF: Molecular Function.


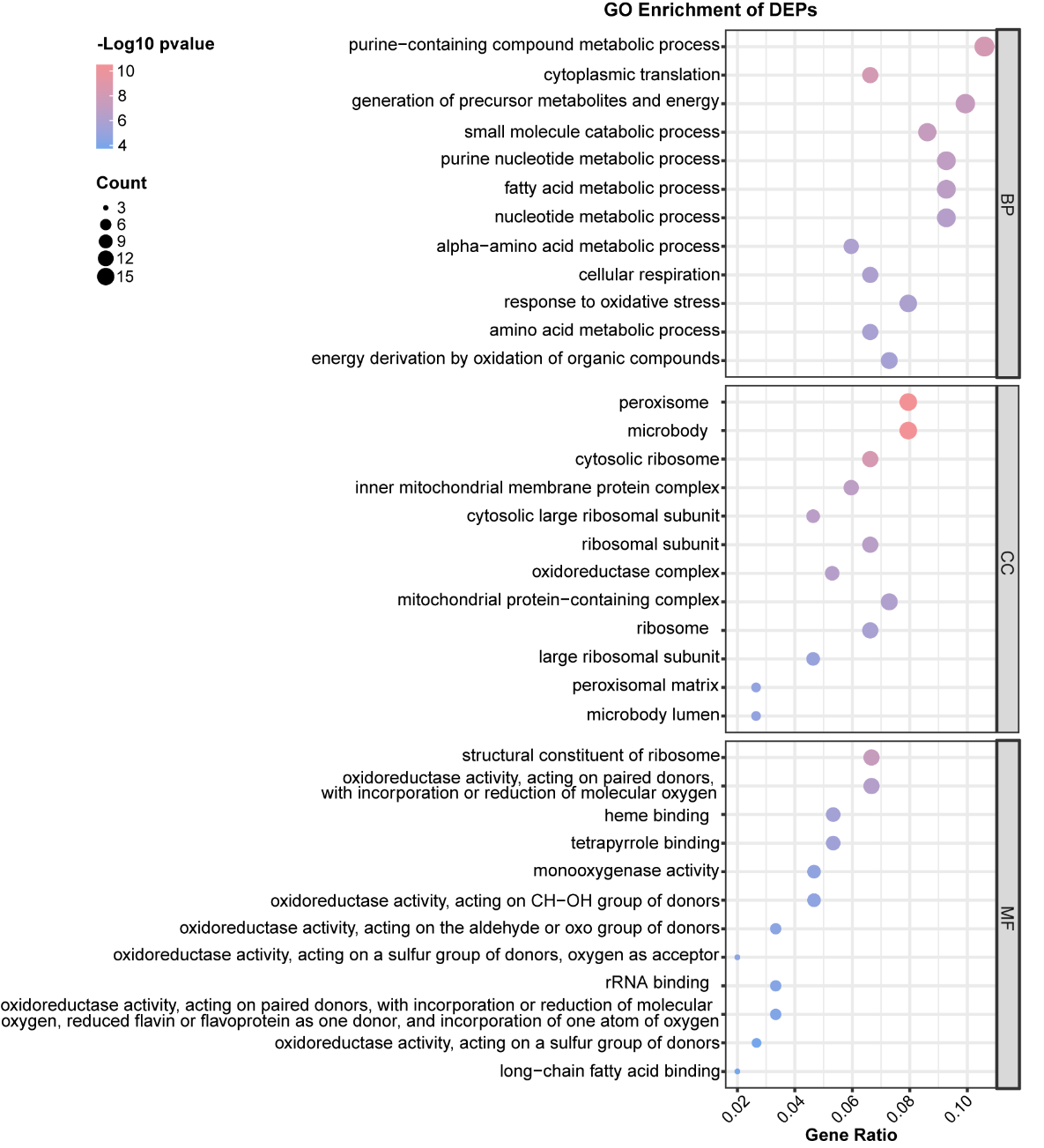


**Fig. S6.** Gene Ontology (GO) terms of differentially expressed proteins (DEPs). BP: Biological Process, CC: Cellular Component, MF: Molecular Function.


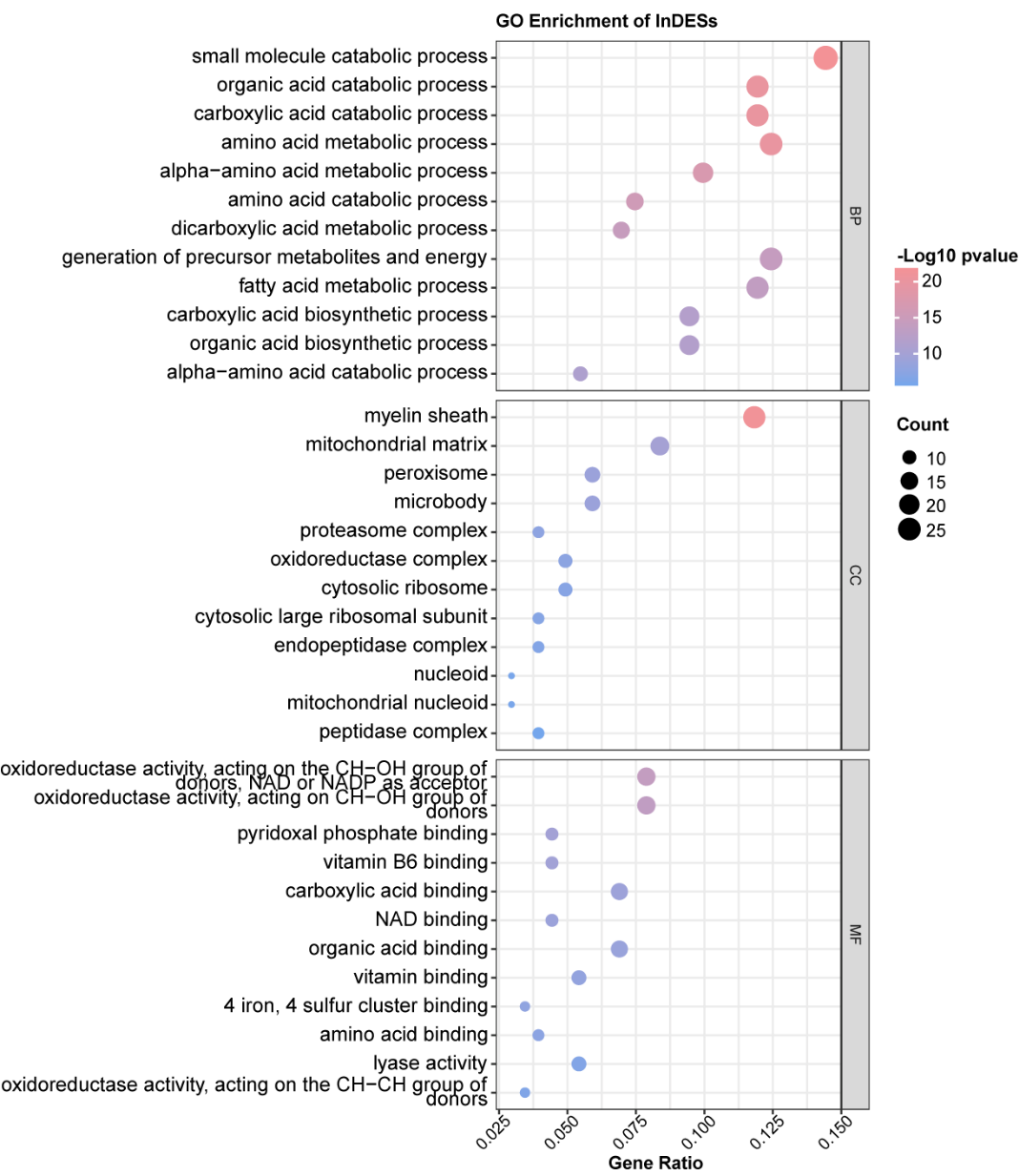


**Fig. S7.** Gene Ontology (GO) terms of independent differential expression N-glycosites (InDESs). BP: Biological Process, CC: Cellular Component, MF: Molecular Function.


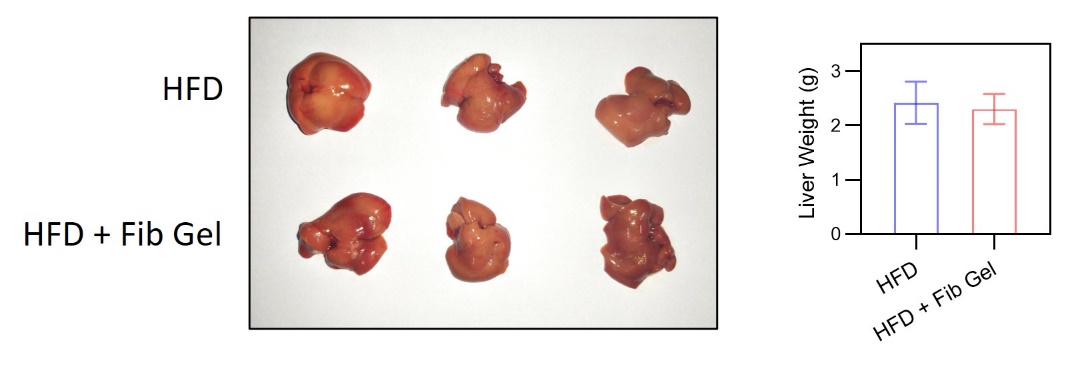


**Fig. S8** Liver images and liver weight of HFD-fed mice treated with drug-free fibrin hydrogel (Fib Gel) every 4 days for 28 days.


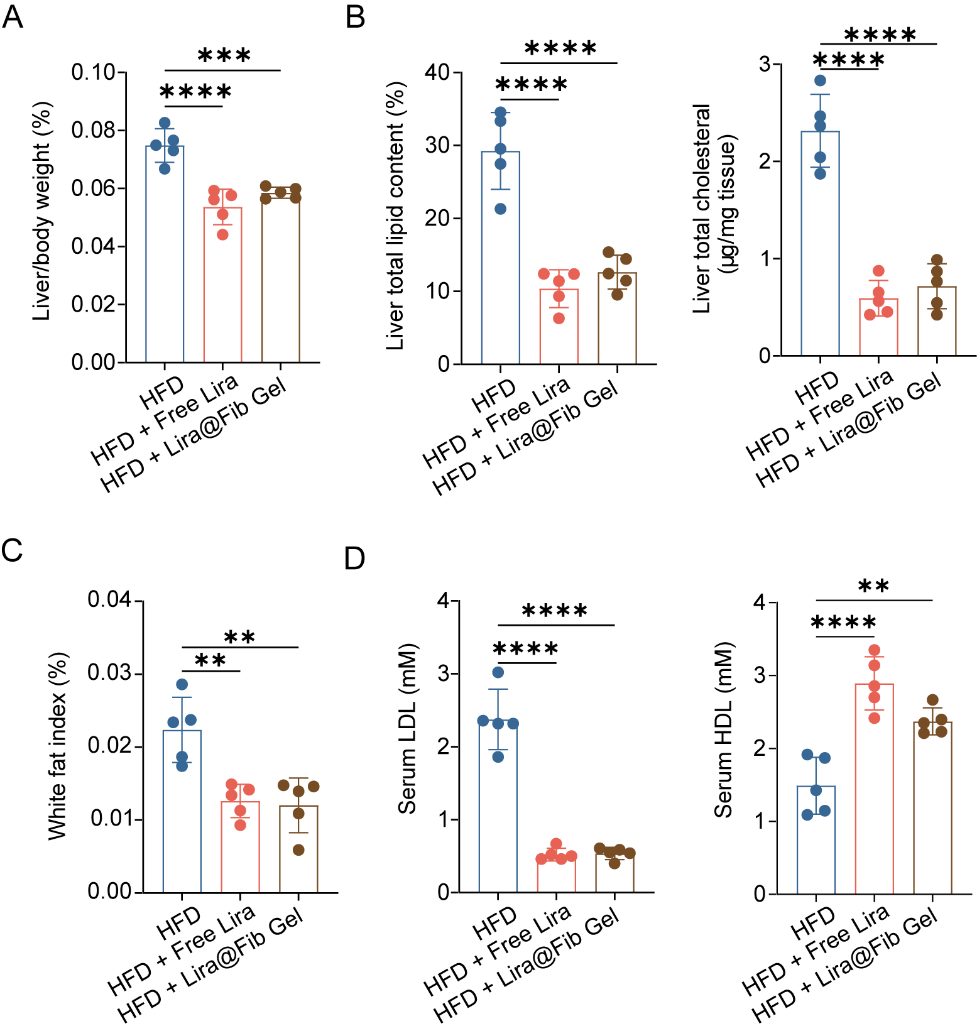


**Fig. S9.** Supplementary data for the therapeutic outcomes of Lira@Fib Gel presented in Figure 6. (A) Liver to body weight ratio, (B) liver content of total lipid and cholesterol, and (C) epididymal white fat to body weight ratio of mice at the study endpoint. (D) Serum level of low-density lipoprotein (LDL), high-density lipoprotein (HDL). *N* = 5 animals per group, data presented as mean ± SD. Statistical significance was calculated using one-way ANOVA with Tukey’s multiple comparisons. ***P*  <  0.01, ****P*  <  0.001, *****P*  <  0.0001, ns: not significant.


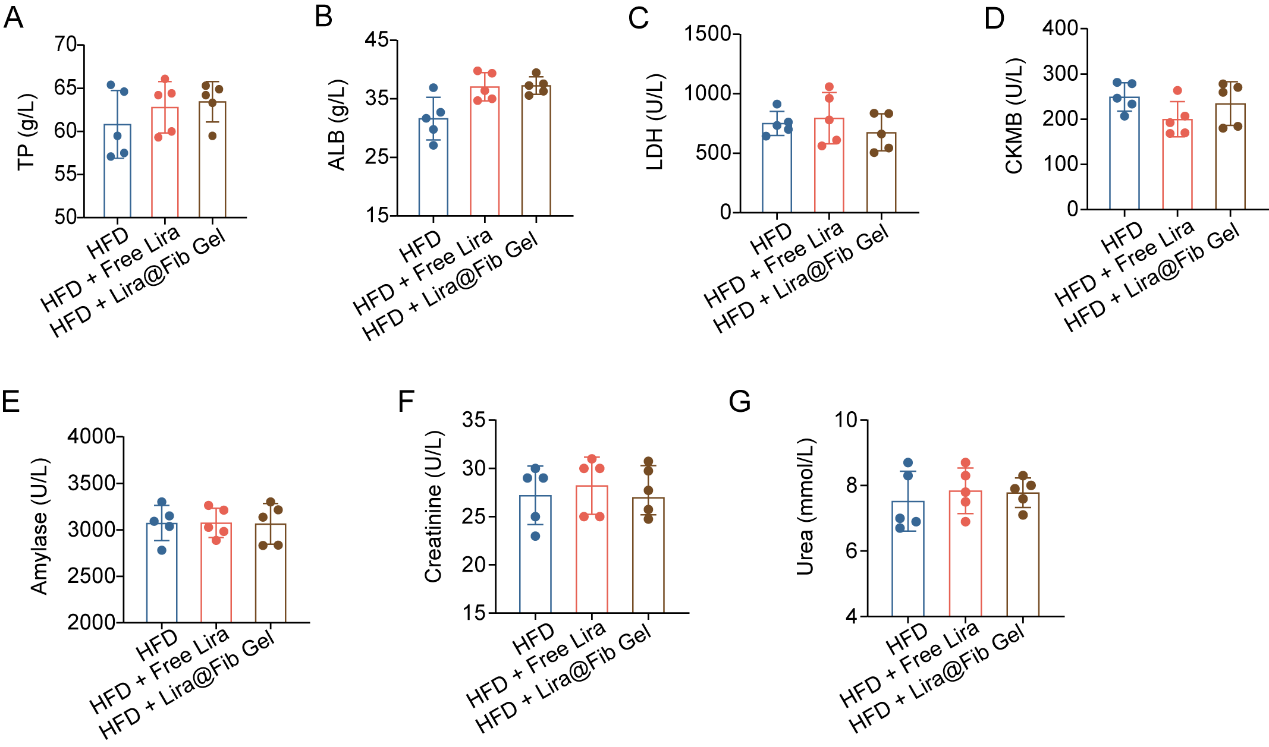


**Fig. S10.** Biosafety of Lira@Fib Gel in the MASLD model C57/BL6 mice after a 28-day therapeutic period. Serum levels of (A) total protein (TP), (B) albumin (ALB), (C) lactate dehydrogenase (LDH), (D) creatine kinase isoenzymes (CKMB), (E) amylase, (F) creatinine, and (G) urea at the study endpoint.


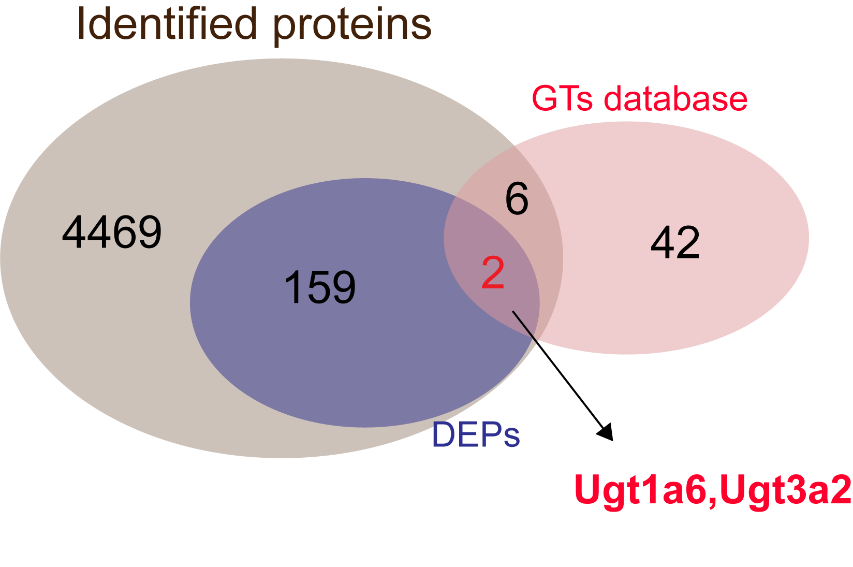


**Fig. S11.** A Venn diagram integrating identified proteins, differentially expressed proteins (DEPs) (HFDL vs. HFD), and glycosyltransferases from the database.
